# Task-related hemodynamic responses are modulated by reward and task engagement

**DOI:** 10.1371/journal.pbio.3000080

**Published:** 2019-04-19

**Authors:** Mariana M. B. Cardoso, Bruss Lima, Yevgeniy B. Sirotin, Aniruddha Das

**Affiliations:** 1 Department of Neuroscience, Columbia University, New York, New York, United States of America; 2 Center for Neural Science, New York University, New York, New York, United States of America; 3 Institute of Biophysics Carlos Chagas Filho, Federal University of Rio de Janeiro, Rio de Janeiro, Brazil; 4 Identity and Data Science Laboratory of Science Applications International Corporation, Annapolis Junction, Maryland, United States of America; 5 Zuckerman Mind Brain and Behavior Institute, Columbia University, New York, New York, United States of America; Vanderbilt University, UNITED STATES

## Abstract

Hemodynamic recordings from visual cortex contain powerful endogenous task-related responses that may reflect task-related arousal, or “task engagement” distinct from attention. We tested this hypothesis with hemodynamic measurements (intrinsic-signal optical imaging) from monkey primary visual cortex (V1) while the animals’ engagement in a periodic fixation task over several hours was varied through reward size and as animals took breaks. With higher rewards, animals appeared more task-engaged; task-related responses were more temporally precise at the task period (approximately 10–20 seconds) and modestly stronger. The 2–5 minute blocks of high-reward trials led to ramp-like decreases in mean local blood volume; these reversed with ramp-like increases during low reward. The blood volume increased even more sharply when the animal shut his eyes and disengaged completely from the task (5–10 minutes). We propose a mechanism that controls vascular tone, likely along with local neural responses in a manner that reflects task engagement over the full range of timescales tested.

## Abstract 2

A neuroimaging study in macaques shows that in the presence of a reward, the task-related hemodynamic neuroimaging response in primary visual cortex becomes more temporally precise and better synchronized with the task.

## Introduction

The use of functional magnetic resonance imaging (fMRI) in humans, complemented with electrode measurements from animal studies, has considerably advanced our understanding of cortical visual processing. This combination of tools has been particularly useful in understanding exogenous, stimulus-evoked responses. Models of neural responses in humans based on electrophysiological recordings in animals, combined with linear models linking neural to hemodynamic responses, have been effective in accounting for stimulus-evoked fMRI measurements in human subjects and in quantitatively predicting the corresponding sensory percepts [1–9].

However, fMRI measurements from subjects performing visual tasks also contain large endogenous hemodynamic responses in the absence of or independent of visual stimuli, even at the earliest stages of visual processing [10–15]. There are at least two types of endogenous response, “attention-like” and “task related” [16]. Unlike the case with exogenous responses, there has been mixed success in interpreting these endogenous hemodynamic responses. Selective visual attention has been characterized extensively through studies in human fMRI [10–15] with close parallels seen in animal electrophysiology [17–25]. Although likely driven by a unified mechanism [26], attention can take different forms. It could be selective for stimulus location [10,11,27–29], features (e.g., color versus motion [30]), or timing [28]. The related hemodynamic responses reflect corresponding attributes of the expected stimuli. Attentional responses also increase in strength along the visual cortical hierarchy [11,20].

Much less is known about the task-related endogenous hemodynamic response, including whether it comprises one or multiple types. It appears to be distinct from selective attention. It entrains to task structure and extends over large sections of cortical areas (e.g., primary visual cortex—i.e., V1) independent of the stimulus [16,31–33], where it can even be substantially stronger than stimulus-selective responses [34]. It is also strongest in V1 and progressively weaker in higher visual areas [16]. These differences may reflect distinct brain processes underlying these two endogenous responses. There is growing evidence of the importance of the task-related endogenous response. It may play a role in sensory processing, in temporally grouping otherwise unrelated sensory stimuli [33] or in switching between stimulus modalities [35]. As yet, relatively little is understood about the mechanism of the task-related response even though its presence has been known for over a decade [16,33,35–41]. This is largely due to the paucity of studies comparing hemodynamics with electrophysiology in behaving subjects.

The current work derives from a task-related hemodynamic response measured using intrinsic-signal optical imaging (ISOI) [42,43] in V1 of behaving macaques performing cued visual tasks [31]. The observed task-related response entrained to task timing independent of visual stimulation, with amplitudes that could compare with or even exceed vigorous visually evoked responses [44]. It appeared to be spatially nonselective, being homogeneous over the optical imaging window and presumably extending beyond [32]. It is thus likely a good model for investigating the mechanism underlying the task-related response seen in humans. Concurrent electrode recordings showed it to be poorly predicted by changes in local firing rates or local field potential (LFP) power at any frequency band [31], unlike stimulus-evoked hemodynamic responses that were well predicted by local electrophysiology [44,45]. Additionally, at a vascular level, this response corresponded to a coordinated contraction–dilation cycle engaging the arterial blood supply into the imaged cortical region [31]. These observations suggested an underlying mechanism distinct from exogenous, stimulus-evoked responses.

Here, we explore the link between this task-related hemodynamic response and the level of engagement in a task. The link was suggested by earlier measurements showing correlations between the measured task-related response and task performance [32], as well as with sympathetic-like markers of mental effort in a task [46] such as phasic pupil dilation [31] and heart rate (HR) fluctuations [31]. To modulate the level of engagement, we changed reward size systematically [47] while the monkeys performed a periodic visual fixation task over several hours. Using ISOI and electrophysiology, we looked for effects on the measured task-related hemodynamic response at multiple timescales: of individual trials (approximately 10–20 seconds), of blocks of trials (150–300 seconds), and finally, of extended segments of task engagement versus disengagement as the animal switched between working and resting with eyes closed (many minutes). Based on our results, we propose that the task-related hemodynamic response reflects mechanisms that entrain brain processing more sharply to a task during periods of higher task engagement, possibly as a means of temporally filtering or binding components of a task. Although we use the term “task engagement” as a shorthand for the set of behavioral and hemodynamic responses described here, in the Discussion, we consider possible links with states of task-specific arousal that have variously been labeled “sustained attention,” “vigilance,” or “alertness” [48–50]. Additionally, we propose an overarching mechanism that controls vascular tone over multiple timescales in coordination with ongoing changes in the level of engagement during a task. Understanding these links would be an important step forward in understanding the dynamic allocation of brain resources in the context of a task.

## Results

### Overview

Two male rhesus macaques performed a cued, periodic visual fixation task, receiving a juice reward following every correct fixation with no time-out or other punishment for errors (see Methods). The task is known to evoke a robust task-related hemodynamic response in the monkeys’ V1 independently of visual stimulation [31,44]. Here, we systematically manipulated the size of the reward per correct trial as a means of modulating the animals’ level of engagement in the task. This was done either in alternating blocks of high and low reward or in sequences of progressively changing reward (see Methods). We recorded V1 hemodynamics using ISOI [42], a high-resolution optical analog of fMRI [51–53]. This technique deduces brain hemodynamic responses at the exposed cortical surface by measuring changes in reflected light intensity at wavelengths absorbed by hemoglobin. Here, we used a wavelength specific to total hemoglobin, which provides a measurement analogous to cerebral blood volume (CBV) [54] (see Methods). Imaging was combined with concurrent extracellular electrode recording of multiunit spiking and LFP. All experimental procedures were performed in accordance with the National Institutes of Health (NIH) Guide for the Care and Use of Laboratory Animals and were approved by the Institutional Animal Care and Use Committees (IACUC) of Columbia University and the New York State Psychiatric Institute.

We observed distinct effects on the task-related V1 hemodynamic responses at the three different timescales tested. At the shortest timescale (individual trials—a few seconds), higher reward led to crisper temporal alignment of the task-related response to each trial, accompanied by a significant, if modest, improvement in response amplitude. At a slower timescale of blocks of alternating high versus low reward (10 to 20 trials—i.e., 150 to 300 seconds per block), we observed consistent alternating ramp-like changes in the mean local cortical blood volume. The sign of the ramps was such as to decrease blood volume for blocks of high reward while increasing it for low. Finally, periods of disengagement from the task during which the animal shut his eyes and rested over many minutes led to further large, sustained increases in the mean local blood volume. None of these effects at any timescale could be accounted for by changes in local spike rate.

The majority of the reported results came from tasks performed in essentially complete darkness (“dark-room fixation” *N* = 30 sites, 3 hemispheres, 2 animals). This allowed measurement of the effects on the endogenous task-related hemodynamic response while minimizing exogenous visual confounds [31]. A complementary section (*N* = 33 sites, 2 hemispheres, 2 animals) confirmed that the observed results generalized to the presence of visual stimuli.

### Timescale of single trials: Higher reward leads to greater temporal precision

**Fig 1:**
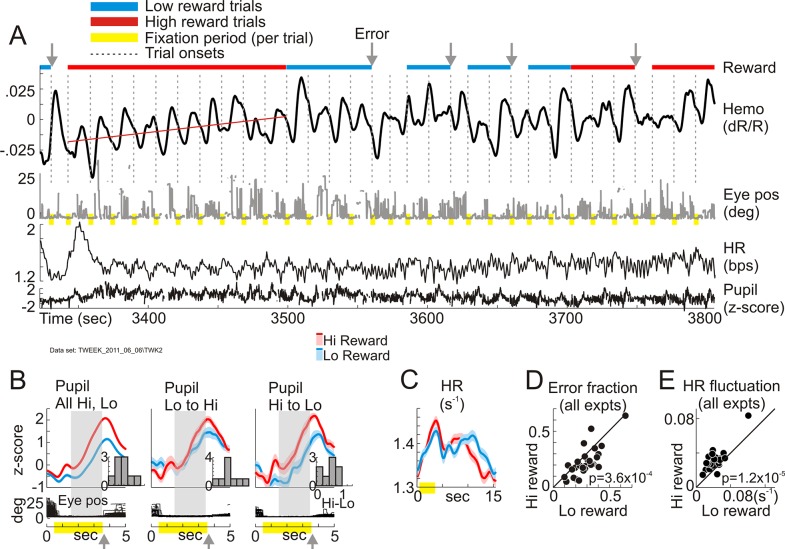
High reward leads to greater engagement in the task. (A-C) Example data set, periodic fixation task in the dark. (A) Continuous records of hemodynamic response (“Hemo”), radial eye position (“Eye pos”), heart rate (“HR”), and pupil size (“Pupil”) while reward level alternated between high (“Hi”; 0.375 ml per correct trial) and low (“Lo”; 0.11 ml) in blocks of 10 correct trials (showing roughly 3 of 32 blocks total, with time indexed relative to the start of the experiment; red = high reward, cyan = low. Same color code is used all through the paper.). Trials with no color indicate incorrect fixation (compare “Eye pos”). Each continuous sequence of incorrect trials counts as one error (gray arrows). Monkeys made more frequent errors in low-reward blocks (0.29 for “Lo” versus 0.19 for “Hi” as fraction of correct trials [*N* = 330]). Hemodynamic response ($\frac{dR}{R}$) plots fractional change in light reflected off cortical surface; down indicates increasing light absorption (i.e., increasing local blood volume). (B) Comparing pupil dilation during the fix period, high- versus low-reward trials. “All Hi, Lo” compares all correct high-reward trials (*N* = 160) with low (*N* = 170). “Lo to Hi” and “Hi to Lo” compare the first trial after a change in reward size to the immediately preceding trial (*N* = 15 “Lo to Hi” transitions, 16 “Hi to Lo” [data in S3 Data]). Gray shaded rectangle indicates a period of steady fixation starting 1 second after fix onset, which is used for quantifying pupil dilation. Inset histograms show dilation difference (high minus low reward) for all experiments with reliable pupil recording (*N* = 9; x-axis labeling, shown only for the third histogram [“Pupil Hi to Lo”] to avoid clutter, is common to all [data in S2 Data]). Rewards were given at the end of each correct fixation (gray arrowheads below time line; the same reward timing was used in all experiments reported here [data in S1 Data]). (C) Comparing amplitude (defined as standard deviation) of mean trial-linked heart rate fluctuations, high versus low reward (0.038 s^−1^: high, 0.025 s^−1^: low [data in S4 Data]). Traces in (B, C) are shown as mean +/− SEM (lighter ribbon). (D) Scatterplot comparing errors as fraction of correct trials, high versus low reward, all experiments (*N* = 30 [data in S5 Data]). (E) Comparing amplitudes of mean HR fluctuation (standard deviation as in panel C), high versus low reward, all experiments (“expts”). Each data point in (D, E) corresponds to one recording site (data in S6 Data). In (D), error trials were counted as high or low reward based on the block in which they occurred (see Methods). In (E), values were averaged separately across all correct high-reward versus correct low-reward trials for the given recording site. *p*-Values in (D and E): Wilcoxon signed rank test.

A section of recording made while the animal fixated periodically in the dark illustrates the pattern of task-entrained responses, as well as changes to these responses with reward size (Fig 1A). Despite the near-total absence of visual stimulation, the V1 hemodynamic recording showed robust task-related fluctuations in local tissue blood volume [31]. These were accompanied, as noted earlier [31], by sympathetic-like responses [46]—i.e., phasic pupil dilation and HR fluctuations—also entrained to the task period. These sympathetic-like responses increased with higher reward. The pupils dilated more per trial, switching dilation size across single trials at block transitions (Fig 1B). The mean HR fluctuations were stronger (Fig 1C and 1E). Furthermore, animals made fewer errors (fixations broken or never acquired) in high-reward blocks (Fig 1A and 1D). The mean hemodynamic response also appeared to ramp slowly upwards during the high-reward block—i.e., reducing mean local blood volume—as indicated by the slope of a linear regression line (red, Fig 1A); this observation is addressed in a later section on slow changes. We did note a weak fluctuation in recorded spiking that was periodic in the mean and appeared to relate to hemodynamics in some data sets. However, the correlation was unreliable and did not generalize (see S1 Fig), consistent with our earlier findings that the task-related response is not predicted by local spiking or LFP [31].

**Fig 2:**
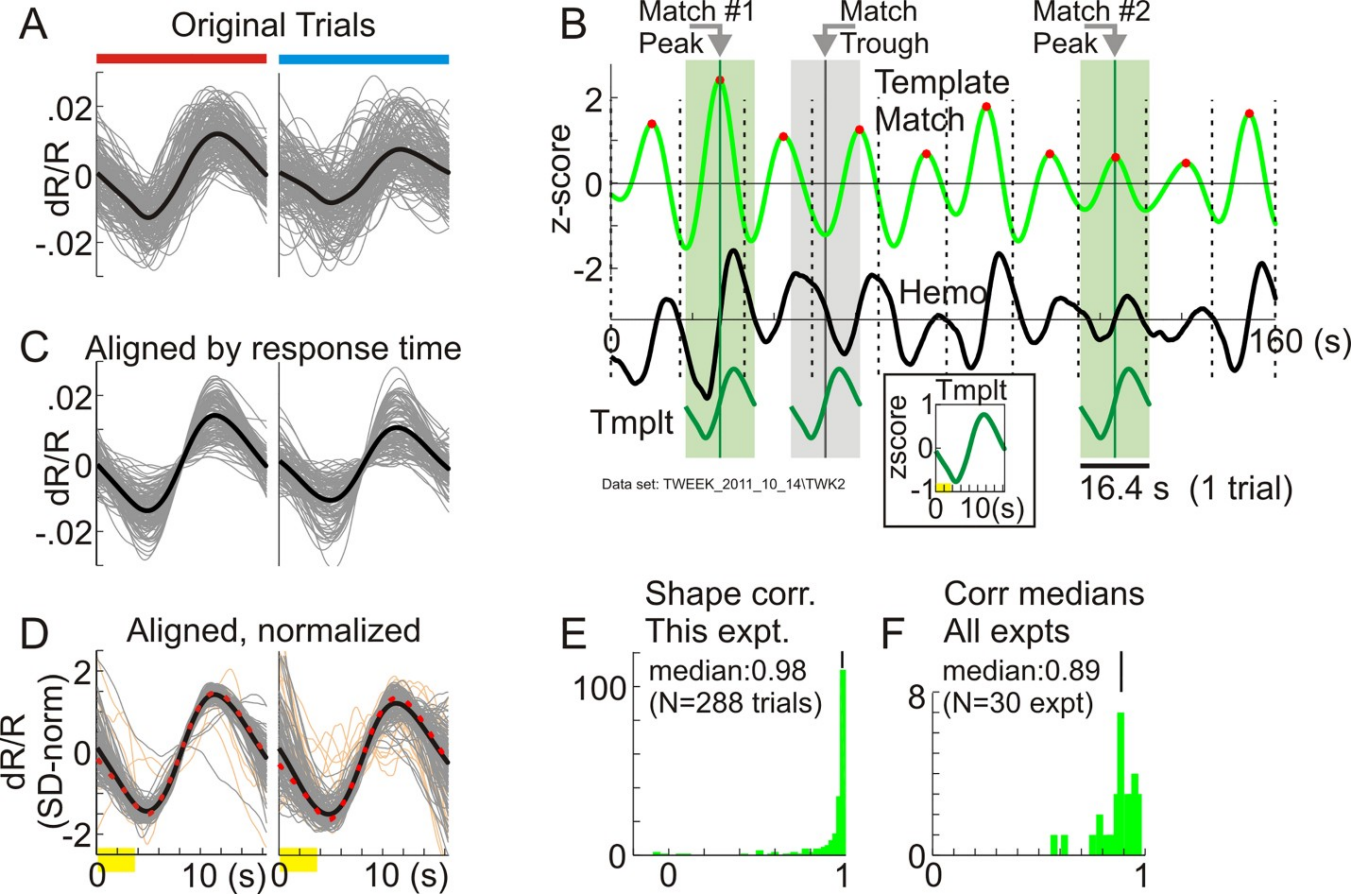
Estimating trial-by-trial timing and amplitude of task-related responses by template matching. (A) All correct trials in one data set, separated by reward size: high (*N* = 140 trials; left, red bar on top as in Fig 1A) and low (*N* = 148, right, cyan bar). Gray indicates individual trials, and black indicates the mean. The time axis is shared with (C), (D) (0 = trial onset; yellow indicates a fixation period [data in S7 Data]). (B) Elements of the template match. Black (“Hemo”) indicates a section of recorded hemodynamic response (z-scored, shifted down for visibility; time indexed from an arbitrary t = 0); vertical dashed lines indicate trial onsets. Green (“Template Match”) marks the sliding-window dot product of “Hemo” with “Tmplt” (inset: defined as mean hemodynamic response, all correct trials). The locations and heights of template match peaks (red dots) define estimated timing and amplitude of task-related response per trial. “Match Peak #1, #2” are examples illustrating the information carried by peaks. Both #1 and #2 mark locations where “Hemo” matches “Tmplt” in shape (see “Hemo” segments on green shading. Compare with “Match Trough,” gray shading, phase-reversed “Hemo”). Greater height of peak #1 versus #2 quantifies higher amplitude of “Hemo” fluctuation at #1. However, location of peak #2 is better centered in its trial. (C) Same traces as in (A), aligned by response times estimated from template match (data in S8 Data). (D) Same data as (C), normalized by amplitude (standard deviation; “SD-norm”). Orange indicates responses with standard deviation in the lowest 10th percentile over the full set. Gray marks the upper 90th percentile. Black indicates the mean of gray traces. The red dotted line marks the template. Gray traces match each other and the template well, particularly near the midpoint of the trial (data in S9 Data). (E) Histogram of correlations (“corr”) of aligned responses with the template (Pearson’s r; all correct trials, high and low reward, including responses in the lowest 10th percentile of standard deviation). (F) Histogram of correlation medians as in (E), all experiments (“expts”; data in S10 Data).

At the timescale of individual trials, the primary correlate of high reward on the task-related response appeared to be greater temporal precision—i.e., tighter alignment to trial timing. This was evident qualitatively in lower trial-to-trial temporal jitter for high reward (Fig 2A, left panel). The mean of these trial-by-trial responses, averaged across all correct trials, was also higher for high-reward trials. But it was unclear how much of that was due to a true difference in amplitude, as opposed to better temporal alignment of individual responses. To resolve this issue, it was necessary to separately estimate the timing and amplitude of the task-related response for each trial.

We used a template-matching approach based on the observation that, other than temporal jitter, individual responses appeared similar to each other in shape independent of reward size (Fig 2A; also see [32]). The full hemodynamic recording was thus modeled implicitly as a sequence of task-related responses of stereotyped shape, one per trial, varying only in amplitude and timing from trial to trial. The template was defined to be the trial-triggered average response over all correct trials. This template was slid in a one-trial-long moving window over the recorded response, calculating the normalized local dot product at each time point (“Template Match” in Fig 2B, Methods, Eqs 1–3). The dot product is closely analogous to Pearson’s correlation (see Methods). We thus surmised that it would have maxima (peaks) at points of high correlation where the recorded hemodynamics locally matched the template in shape—in effect, defining locations of putative task-related responses. But in addition, unlike Pearson’s r, which is scale-invariant, dot products scale linearly with the amplitude of their arguments and thus provide a measure of response strength (Fig 2B and Methods). We therefore defined our estimates of task-related response time and amplitude per trial to be the location and height of the corresponding template match peak.

After estimating response times and amplitudes as described above, we wanted to check our starting assumption that the measured hemodynamics are well modeled as a sequence of jittered but stereotyped shapes. If the assumption is valid, the segments of recorded hemodynamics centered on each peak of the template match should match each other closely in shape. To test, we centered each putative task-related response, as picked out through template matching, by its response time as estimated from the same template match (Fig 2C). Indeed, the realigned responses were strikingly well correlated with each other. This can be appreciated visually by normalizing realigned responses by their amplitudes to help compare shapes (Fig 2D) and quantitatively by correlating realigned responses to the template used for matching (Fig 2E and 2F). The strength of this correlation supports our approach.

**Fig 3:**
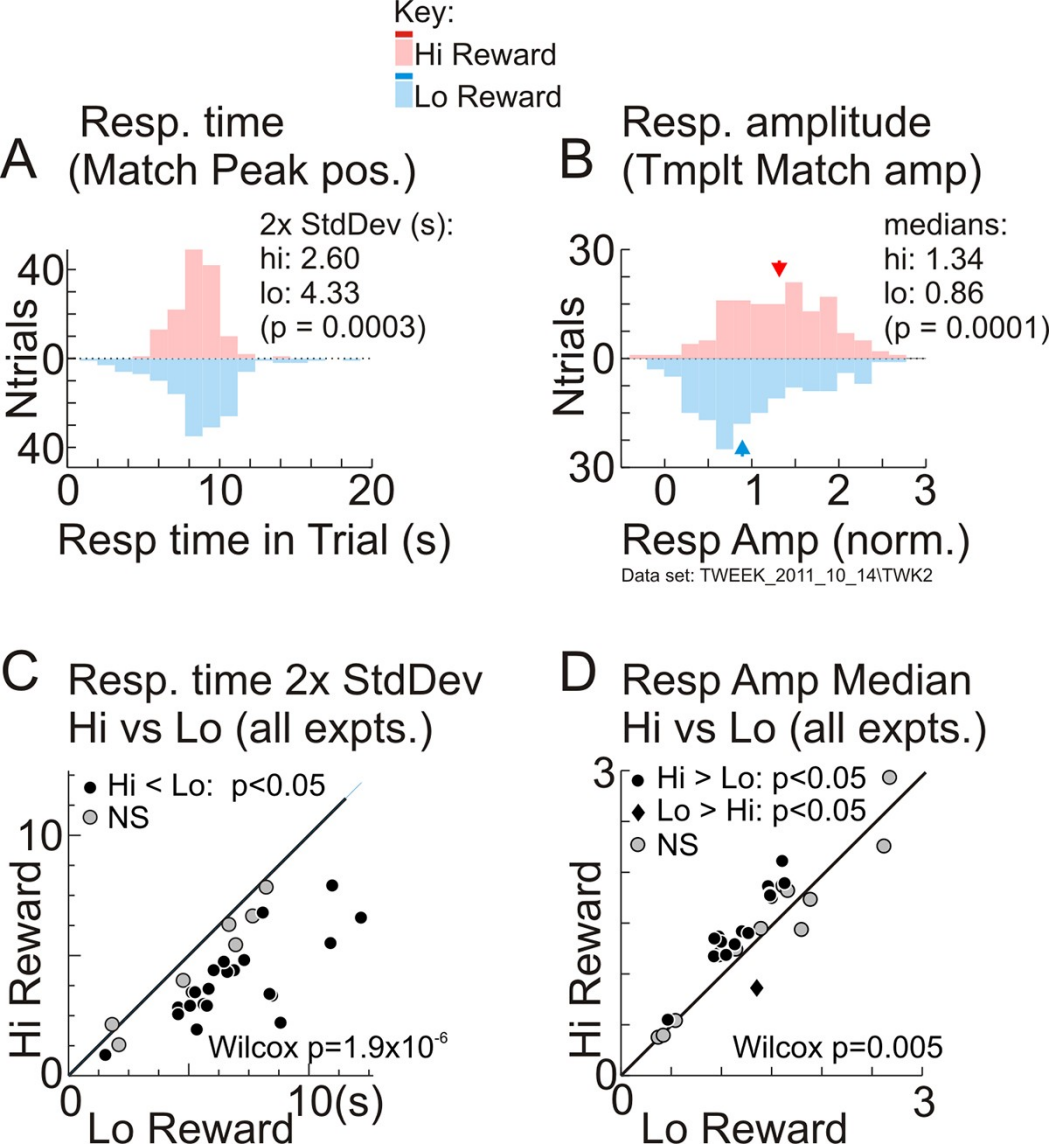
Higher reward leads to more temporally precise task-related responses. (A) Distributions of response (“Resp.”) times per trial, estimated as positions of the corresponding template match peaks (“Match Peak pos.”), same data set as in Fig 2A–2E. Distributions are separated by reward size, with color coding as indicated in the key (common to (A, B) and to all later figures). Data are shown as a vertical “violin plot” histogram with numbers of trials increasing from 0 (middle) upwards for high reward (“Hi”) and downwards for low (“Lo”). Similar displays are used for all such comparisons of distributions to avoid clutter (e.g., with interleaved histograms). Clustering of response times per reward size was quantified as the 2 standard deviation (“2x StdDev”) width of timing distributions. (B). Distributions of response amplitudes per trial, estimated from template matching (“Tmplt Match”), shown separated by reward size following the same conventions as in (A). Response amplitude per reward size was quantified as the median (indicated by arrowheads; medians are indicated similarly in all later amplitude distributions). Significance (*p*-values) in (A,B) were obtained from bootstrap with 10,000 resamples. (The 2x StdDev values per reward size [“hi”,”lo” in panel A] and median amplitude per reward size in panel B shown in these and other panels are not the sample medians from the measured distributions but rather are medians obtained from the same bootstrap procedure used to get *p*-values.) (C) Comparing the 2 standard deviation width of the response time distribution for all correct high-reward trials versus that for all correct low-reward trials, per experiment (“expt”; *N* = 30). (D) Comparing median response amplitudes for all correct high- versus all correct low-reward trials, per experiment. *p*-Values in (C), (D): Wilcoxon signed rank tests for pairwise comparisons (data in S11 Data).

With the task-related response times and amplitudes thus quantified, we confirmed that the primary effect of higher reward was greater temporal precision. Response times were better aligned to the task period, with consistently tighter distributions (quantified by the 2 standard deviation width of the distribution). This was evident for the example data set (Fig 3A) as well as in essentially every other data set (Fig 3C). High reward also led to significantly higher response amplitude for the example data set (Fig 3B). However, that pattern was less consistent over the full set of experiments, with only a relatively modest improvement in median response amplitudes overall (Fig 3D).

We wondered if these results were due to our particular choice of template. We tested by repeating the analysis shown here across all data sets using a range of alternate templates. The alternate templates were also each one trial long and constructed from measured responses but using different criteria: for example, being phase shifted in time or using only “high signal-to-noise” responses with amplitudes exceeding a threshold. The task-related response times and amplitudes estimated by matching to these alternate templates showed a strikingly similar overall relationship to reward size as in Fig 3. This is illustrated in S2 Fig for a particular alternate template with timing and shape distinct from the one used in the main text. This result highlights the overall robustness of our findings. It also suggests that high reward leads to a state of greater temporal regularity and periodicity overall for the duration of the block, accounting for the higher temporal precision in estimated response times independent of the details of the template used.

### Timing precision is robust to noise in template match

**Fig 4:**
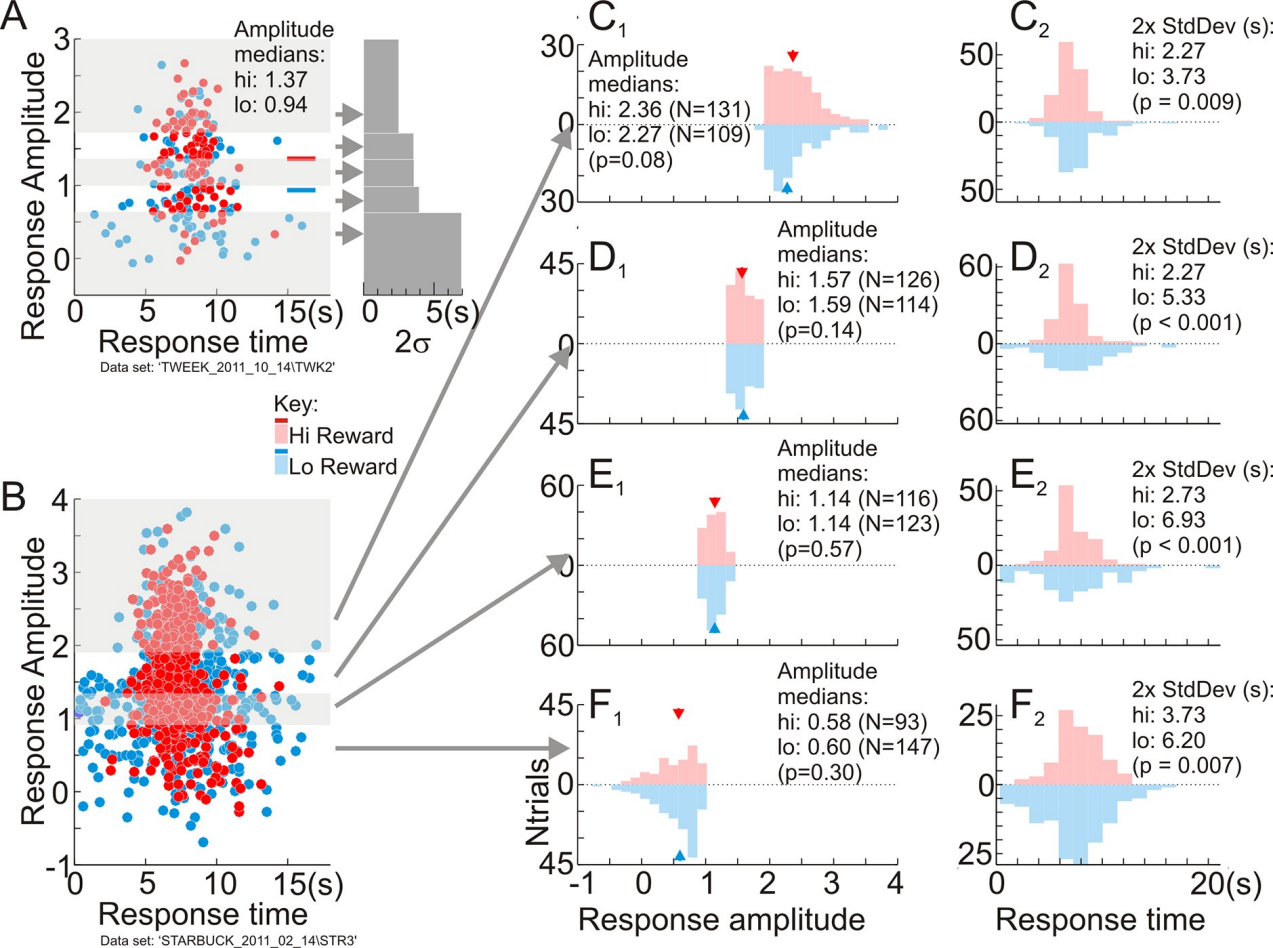
Temporal precision is not an artifact of higher signal for high-reward responses. (A) An outline of the null hypothesis using a data set in which low-reward responses had substantially lower amplitudes. Left panel: Scatterplot of response amplitude versus time per trial, colored by reward size. Gray and white shading indicates quintiles along the amplitude axis, combining high (“hi”) and low (“lo”) rewards. Right panel: 2 standard deviation width of response time distribution in each quintile. The time axis is scaled to match that for the left panel. These 2 standard deviation widths (“2x StdDev”) increased progressively for lower response amplitudes, which were also more dominated by low-reward trials. The null hypothesis is that this covariance alone gives low-reward trials larger timing scatter. Arrows mark the y-axis locations indicating median amplitudes of quintiles (data in S12 Data). (B) Plot of response amplitude versus timing for a large data set (1,285 correct trials; 629 low reward; 626 high reward) separated into quartiles by response amplitude (gray/white shading). Each quartile also roughly matched for numbers of low- versus high-reward trials (data in S13 Data). (C1, C2, D1, D2, E1, E2, F1, F2) Pairs of distributions of response amplitude and timing per quartile separated by reward size. The numbers “N” in parentheses in (C1–F1) indicate numbers of high- and low-reward trials. The high-reward responses are significantly more precise than the corresponding low-reward ones in each quartile despite the similarity in response amplitudes (C2–F2). All distributions are shown as “violin plots” using the same conventions as Fig 3A and 3B. Panels (C1–F1) share a common abscissa scale, as do panels (C2–F2). The “NTrials” label for the ordinate is shown only for (F1) to avoid clutter. *p*-Values, bootstrap, 10,000 resamples (data in S14 Data).

We were concerned that the apparently lower temporal precision with low reward could be an artifact of a noisier template match. When task-related responses had lower amplitudes, the template match could be poorer simply because of lower signal to noise. This could lead to noisier estimates of response time with wider distribution and thus apparently poorer precision but, because of the poorer signal to noise alone, independent of reward size (Fig 4A; also consider, e.g., the responses with poor shape match in Fig 2D). Since lower rewards were associated with somewhat lower response amplitudes, this increased noise could make the low-reward responses appear artifactually less precise.

To test, we selected subsets from each experiment in which the high- and low-reward responses were matched in amplitude and in numbers of data points (Fig 4B, 4C1, 4D1, 4E1 and 4F1). If our concern about signal to noise in the template match were valid, high- and low-reward responses in each amplitude-matched subset should exhibit similar distributions of response times independent of reward size. Instead, even after matching for amplitudes, the high-reward responses remained consistently and significantly more temporally precise (see, particularly, Fig 4D1, 4D2, 4E1 and 4E2).

### Timing precision is independent of eye fixation timing and eye movements

We wondered if there were some simple oculomotor explanation or correlate of our observation. We considered two possible scenarios under which this could happen.

We considered the null hypothesis that the timing of the task-related response per trial is determined by fix onset, with a stereotyped response time course and hence constant delay following fixation (see S3 Fig). If that were the case, response times should be correlated to fix onset times, with unity slope and constant delay. The higher precision with high reward could reflect a behavioral pattern in which the animal is more precise in its fix onsets prior to those trials (S3 Fig, panel a). This null hypothesis turned out not to be the case, and task-related response times were uncorrelated with fixation onset. Parenthetically, both animals’ fixation behavior changed over the many months that we tested them intermittently on this task. Initially, both animals tended to maintain fixation for long periods with very few breaks even during intertrial intervals. This led to extended periods of fixation prior to the start of each trial, or even across multiple trials, without any breaks but unrelated to the timing of the task-related response or reward size (S3 Fig, panel b). Later, both animals showed a different behavioral pattern, moving their eyes around during intertrial intervals and reacquiring fixation shortly before trial onset. This led to a pattern of brief fixation periods prior to each trial (S3 Fig, panel c). Task-related hemodynamic response times remained more precise with high reward, independent of the changing pattern of fixation.

We next considered the possibility that animals may have steadier fixation or smaller eye movements during high-reward blocks, due to generally higher engagement in the task (S4 Fig). We failed to see any consistent patterns. There were no consistent differences in fixational jitter between high- and low-reward trials at the resolution of our measurements (60 Hz, 0.33 deg). There were also no consistent differences in eye movements during the intertrial periods during which the animals were free to look around. The animals also changed their patterns of intertrial eye movements over the many months of recording. In earlier sessions, they did move their eyes less during high-reward blocks (S4 Fig, panels a1-a3). Later, however, the animals adopted a behavioral pattern of greater intertrial eye excursions for high-reward trials (S4 Fig, panels b1-b3). However, the task-related responses remained more precise for higher-reward trials (smaller 2 standard deviation width for task-related response time distributions), independent of this changing pattern of eye movements.

### Timing precision generalizes to the presence of visual stimulation

**Fig 5:**
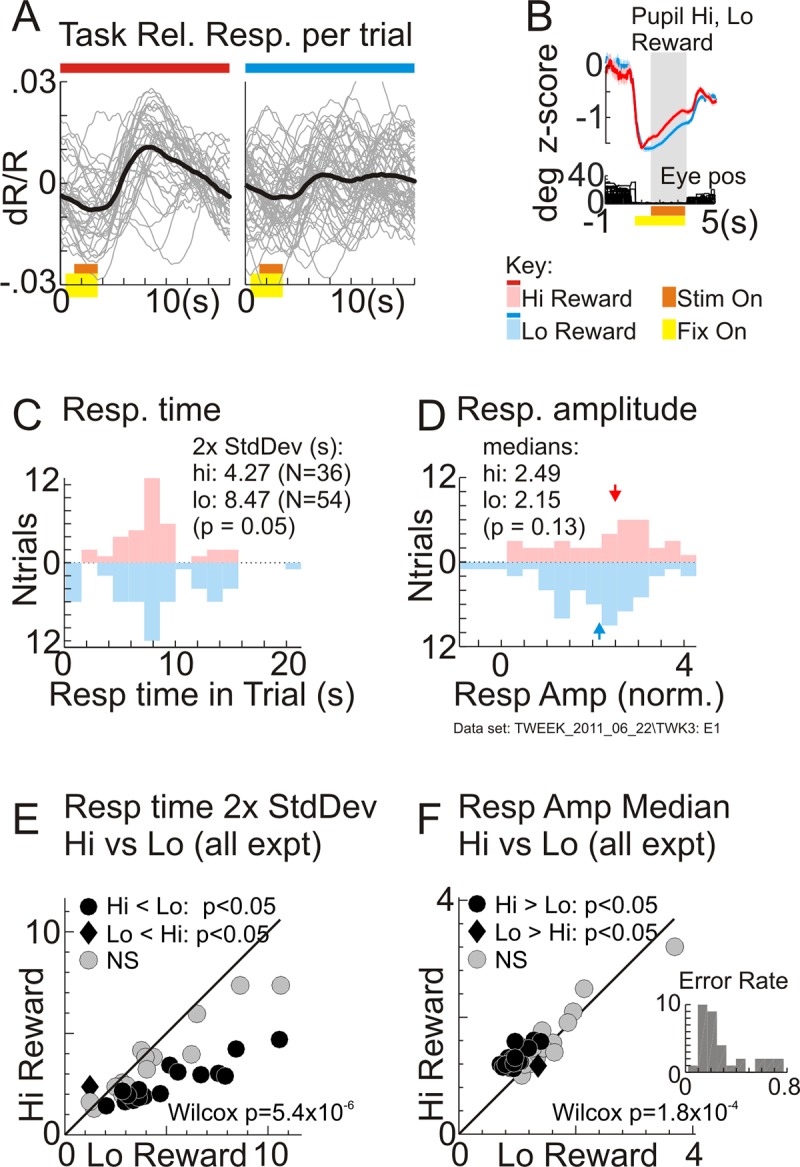
Task-related responses in the presence of visual stimuli are temporally more precise and modestly higher in amplitude for high reward as in the dark room. (A-D) One example data set: (A) Residual task-related responses (“Task Rel. Resp.”) separated by trial and by reward size were obtained by regressing away stimulus-evoked responses (see S5 Fig) (data in S15 Data). (B) Comparing pupil dilation for high- (“Hi Reward”) versus low-reward (“Lo Reward”) trials. Gray shading indicates the period over which pupil dilations are compared, starting 1 second after fixation. (These pupil measurements were made in the presence of visual stimulation, unlike dark-room results [Fig 1B], likely accounting for different shape of trace including initial constriction on fixation) (data in S16 Data). (C) Distribution of response times from template match in this data set. (D) Distribution of response amplitudes in this data set. Conventions for “violin plot” histograms are used as in Fig 3A and 3B (data in S19 Data). (E) Comparing the 2 standard deviation widths of response times (“Resp time 2x StdDev”) for high versus low reward, per experiment (“expt”; *N* = 33) (data in S17 Data). (F) Comparing median response amplitudes (“Resp Amp Median”) for high versus low rewards, per experiment (*N* = 33). *p*-Values in (E), (F): Wilcoxon signed rank tests for the pairwise comparisons. The inset in panel F indicates overall behavioral performance as the total numbers of error trials as a fraction of the correct trials, per experiment (data in S18 Data). Eye pos, eye position; Fix on, fixation on; norm., normalized; NS, not significant; Stim on, stimulus on.

The question that remained was whether reward size affected task-related responses only in the unnatural circumstance of visual tasks in the near absence of all visual stimulation or whether such effects generalized to the presence of visual stimuli. To test, we analyzed data from a separate set of experiments in which the animals were passively shown visual stimuli—gratings of different contrasts—while performing the same cued, periodic fixation task (Fig 5). Rewards were comparable to the dark room, if slightly higher (see Methods), ranging typically from 0.2 ml/trial (low) to 0.6 ml/trial (high). For this, we first needed to estimate the task-related response from recorded hemodynamics by estimating and removing stimulus-evoked responses. We did so by modeling the overall measured hemodynamics as a linear sum of the stimulus-evoked and task-related components, which we fitted to get the optimal kernels for the two components[55] (Methods, Eqs 4–6; also, S5 Fig). The optimal hemodynamic response function (HRF) kernel thus obtained was then convolved with the recorded spiking to estimate the stimulus-evoked component of hemodynamics and regress it away from the full hemodynamics. The residual—that was, by construction, the component of hemodynamics not predicted by local spiking—was then defined to be the task-related component of the hemodynamic response, equivalent to the full hemodynamic response in the dark room.

The task-related response thus estimated in the presence of visual stimuli was again temporally more precise with high reward, just as with the task undertaken in the dark room. This can be seen qualitatively after separating the estimated task-related response into individual trials and segregating trials by reward size. These trial-wise responses were visibly less temporally jittered for high reward (Fig 5A). The timing and amplitude of these task-related responses were quantified by matching to a template just as for recordings in the dark room; the template was taken to be the optimal mean kernel for the task-related component as estimated from the fit (Methods, Eqs 7 and 8. S5 Fig). The results of this template match closely paralleled those obtained in the dark room fixation task. The estimated response times were again more tightly clustered for high-reward trials, both for this specific data set (Fig 5A and 5C) and over the set of visually stimulated experiments (Fig 5E). Response amplitudes showed only a modest improvement (Fig 5D and 5F). High-reward trials were also associated with greater pupil dilation (Fig 5B).

### Timescale of blocks of trials: Mean blood volume decreases for high reward and increases for low

**Fig 6:**
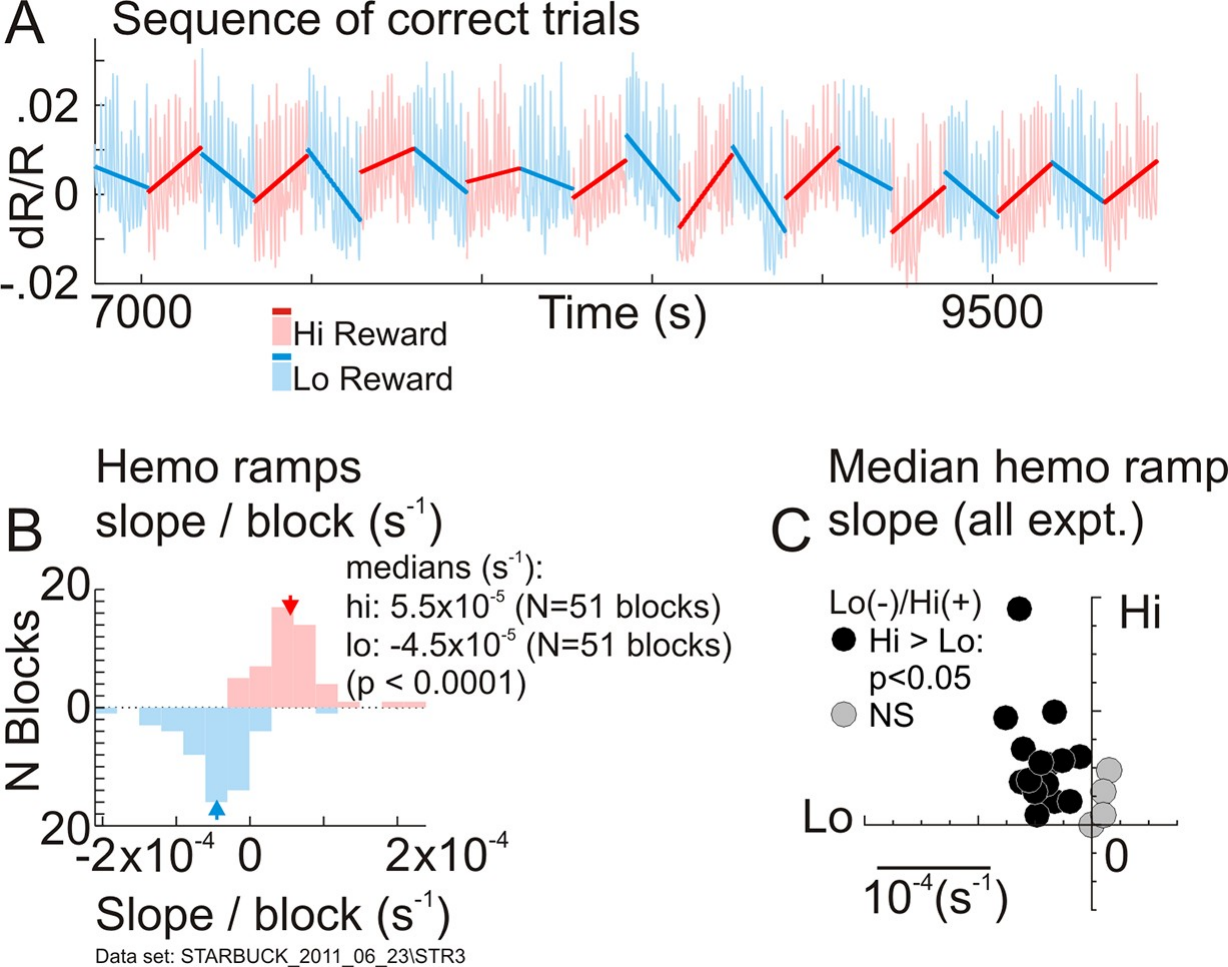
Mean local cortical blood volume increases for low-reward blocks and decreases for high, in alternating ramp-like drifts. (A) Recordings from a sequence of correct trials, alternating between high (“Hi Reward”) and low reward (“Lo Reward”) in blocks of 10. Lines indicate regression fits to each block separately. Increasing slope implies decreasing local tissue blood volume. Correct trials were concatenated after excising incorrect ones while maintaining vertical position (see S6 Fig). This panel shows 20 blocks of 102 total: 1,160 trials total, 1,019 correct. (B) Histogram of regression slopes in high- versus low-reward blocks. Same data set as (A) (*p*-values, bootstrap, 10,000 resamples) (data in S21 Data). (C) Comparing median slopes of high-reward versus low-reward blocks over the set of all experiments (“expt.”). All the statistically significant data points lie in the upper left quadrant, “Lo(-)/Hi(+)”—i.e., with negative slopes for low- and positive slope for high-reward blocks (*N* = 19 experiments: using only those with at least 10 pairs of alternating blocks of 10 trials each). The results shown here were based on correct trials alone. Analyses that utilized all trials including incorrect ones gave results that were broadly similar but were sometimes harder to interpret because of the arbitrary duration of sequences of incorrect trials (S6 Fig) (data in S20 Data). Hemo, hemodynamic response; NS, not significant.

Analyses up to this point were restricted to the scale of single trials—i.e., about 10 to 20 seconds. However, we also noted slow ramp-like drifts in the mean local blood volume over blocks of 10 to 20 trials of a given reward size—i.e., about 150 to 300 seconds (Fig 1A; Fig 6). The ramps decreased blood volume for high-reward blocks while increasing it for low. Regression lines fitted through sequences of correct trials per block clustered into distinct sets of negative slopes (increasing absorption of light during imaging—i.e., increasing blood volume) for low-reward blocks and positive slopes (decreasing blood volume) for high (Fig 6B and 6C).

These slow hemodynamic drifts were not driven by slow changes in spiking (see S7 Fig). Blocks of trials with alternating ramps of mean blood volume failed to show similar alternating ramps of mean spiking (S7 Fig, Panels a-d). To test more quantitatively, we first simulated the spiking patterns required to generate the measured hemodynamic slopes on convolving with the corresponding optimal fitted HRF per experiment (S7 Fig, Panels e, f). The slopes of the simulated spiking ramps alternated in sign with reward size, as expected. Each measured spiking slope was then divided by the slope of its corresponding simulation to compare. If the measured slopes had the same sign as their simulations, these ratios would consist of positive numbers, with some magnitude reflecting a scale factor. This was not the case; the ratios were equally likely to be positive or negative for both high and low reward. The measured spiking slopes were thus uncorrelated with those required to generate the measured hemodynamic slopes.

### Switching from task engagement to rest with eyes closed: Further profound increases in blood volume

**Fig 7:**
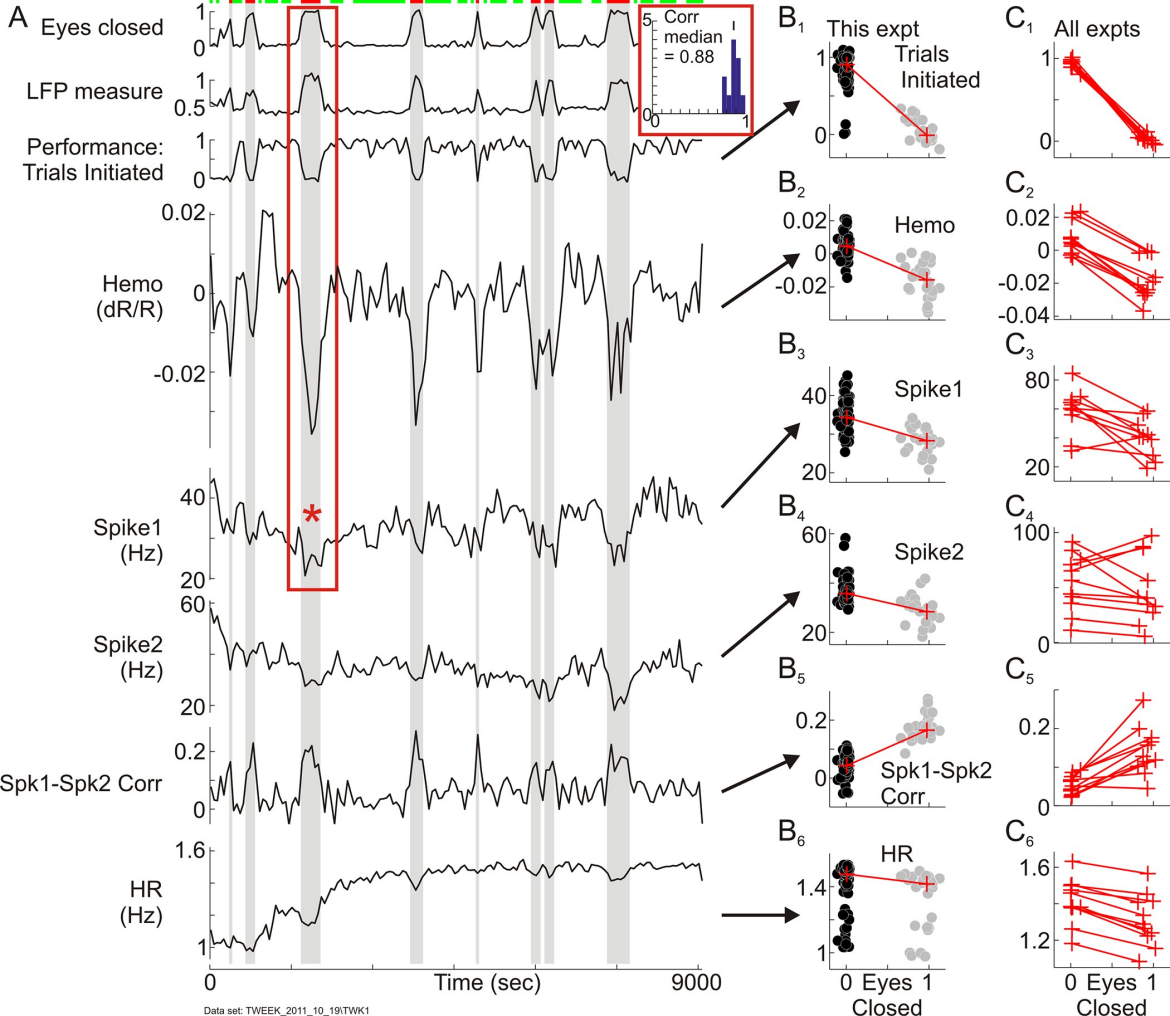
Mean local blood volume, spiking, and heart rate trace switches between states of task engagement and rest. (A) Traces show continuous 2.5-hour records of measured variables as indicated by adjoining labels, smoothed and downsampled to a 60-second sampling rate to track slow changes. (See text and Methods for more details.) Red and green bars on top mark epochs of rest (defined as “Eyes closed” > 0.6—i.e., >60% of the 60-second sample time) and task engagement (sections with eyes open, defined as “Eyes closed” < 0.05—i.e., <5% of the time). “Eyes closed” is highly correlated with the LFP measure (see text for definition. Pearson’s r = 0.94 for the example data set. Inset shows histogram of Pearson’s r for similar pairwise correlations over all data sets used for this analysis, *N* = 11). Performance in the task is quantified as the (smoothed) fraction of trials initiated in the 60-second (i.e., approximately 4-trial) window. “Hemo” marks the mean hemodynamic response (dR/R); “Spike1” and “Spike2” mark multiunit responses recorded from two electrodes spaced 4 mm apart in the imaged region. “Spk1-Spk2 Corr” marks the pairwise correlation between these two recordings over a 1-second moving window. “HR” marks mean heart rate. The red box indicates the section of hemodynamic and corresponding Spike1 spiking measurements (red asterisk) that are analyzed at a higher temporal resolution in Fig 8A. (B1-B6) Scatterplots of the measured values for the given experiment, as indicated, versus “Eyes closed.” Each data point represents a single smoothed, nonoverlapping 60-second sample. Data points are segregated into “task-engaged” (black) and “rest” (gray) using the value of “Eyes closed” as described. Red lines connect medians (data in S22 Data). (C1-C6) Lines connecting medians as in (B1-B6) for all experiments (“expts”) used (*N* = 11) (data in S23 Data). LFP, local field potential.

We wondered if the slow increase in mean local blood volume accompanying reduced reward could be part of a broader pattern of shifts in mean local blood volume accompanying shifts in the level of engagement. A potential clue was seen in the continuous measurements during long dark-room recording sessions lasting up to 3 hours (Fig 7). In these sessions, in between extended stretches of working well, the animals would take occasional breaks of many minutes during which they stopped working and rested with their eyes shut. The mean local blood volume in V1 increased strikingly during these breaks, returning to baseline when the animal resumed working (Fig 7A). This pattern appeared to be an extreme manifestation of the ramp-like changes in blood volume with reward size in which lower reward, with its lower level of engagement (Fig 1), led to increasing mean blood volume (Fig 6).

Before ascribing an association with reduced engagement in the task, we needed to test whether the increased blood volume could be accounted for simply by concurrent changes in neural or physiological drivers. As possible drivers, we considered the mean HR and the mean local multiunit spike rate (recorded separately at two electrodes spaced 4 mm apart in the recording chamber). We also measured the pairwise noise correlation of spike rates between the two electrodes over a 1-second sliding window, since that was expected to increase at rest [56]. To focus on slow changes, all recordings were downsampled to get, in effect, the smoothed average in a 60-second window (see Methods).

We then assessed changes in these physiological and neural measurements as the animal switched state, marking the state based on the fraction of time within the 60-second window that the eyes were closed (Fig 7A, top row, “Eyes closed”). Spontaneous eye closures in the dark have been shown to provide a useful measure of drops in “vigilance” [49], correlating well with electroencephalographic (EEG) and fMRI indicators [57]. For this study, epochs during which the eyes were shut more than 60% of the time were defined as “rest,” whereas those with less than 5% of time with eye closure were considered “engaged.” To check, we compared with an LFP measure of arousal based on the ratio of power in the beta- and theta-range frequency bands (15–25 Hz and 3–7 Hz, respectively) as suggested by earlier studies ([57]; also reviewed in [48]). To get a measure that was low when the animal was engaged in his task and high when at rest [48], as with eye closure, we placed the theta power in the numerator. The square root of this ratio further compressed the dynamic range to roughly 0–1, as with eye closure. These two measures based on eye closure and on the LFP were closely comparable (Fig 7A, upper two rows and inset), supporting the use of eye closure to segregate physiological measurements by the state of task engagement.

The mean neural and HR measurements thus segregated showed systematic changes as the animal switched between states of rest and task engagement but in a direction opposite to that expected to increase blood volume at rest (Fig 7A). Thus, the mean HR, averaged over the moving 60-second window, reduced systematically relative to its local baseline value each time the animal disengaged from work and rested with eyes shut (Fig 7A: bottom trace [“HR”]: see shaded areas indicating rest). This is consistent with the abrupt falls in mean HR and blood pressure seen at sleep onset in human subjects [58]. But it suggests that the concurrent increase observed in V1 local blood volume is not a passive consequence of cardiovascular changes, as that would require an increase rather than a decrease in HR [59]. Similarly, the mean spike rate recorded at individual electrodes typically decreased as the animal rested. If the blood volume at rest were driven linearly by local spiking, then the mean spike rate should have increased [1]. Although the mean spike rates at individual electrodes largely decreased, the pairwise correlation of spike rates over the pair of electrodes showed the expected [56] striking increases for the epochs of rest versus engagement.

**Fig 8:**
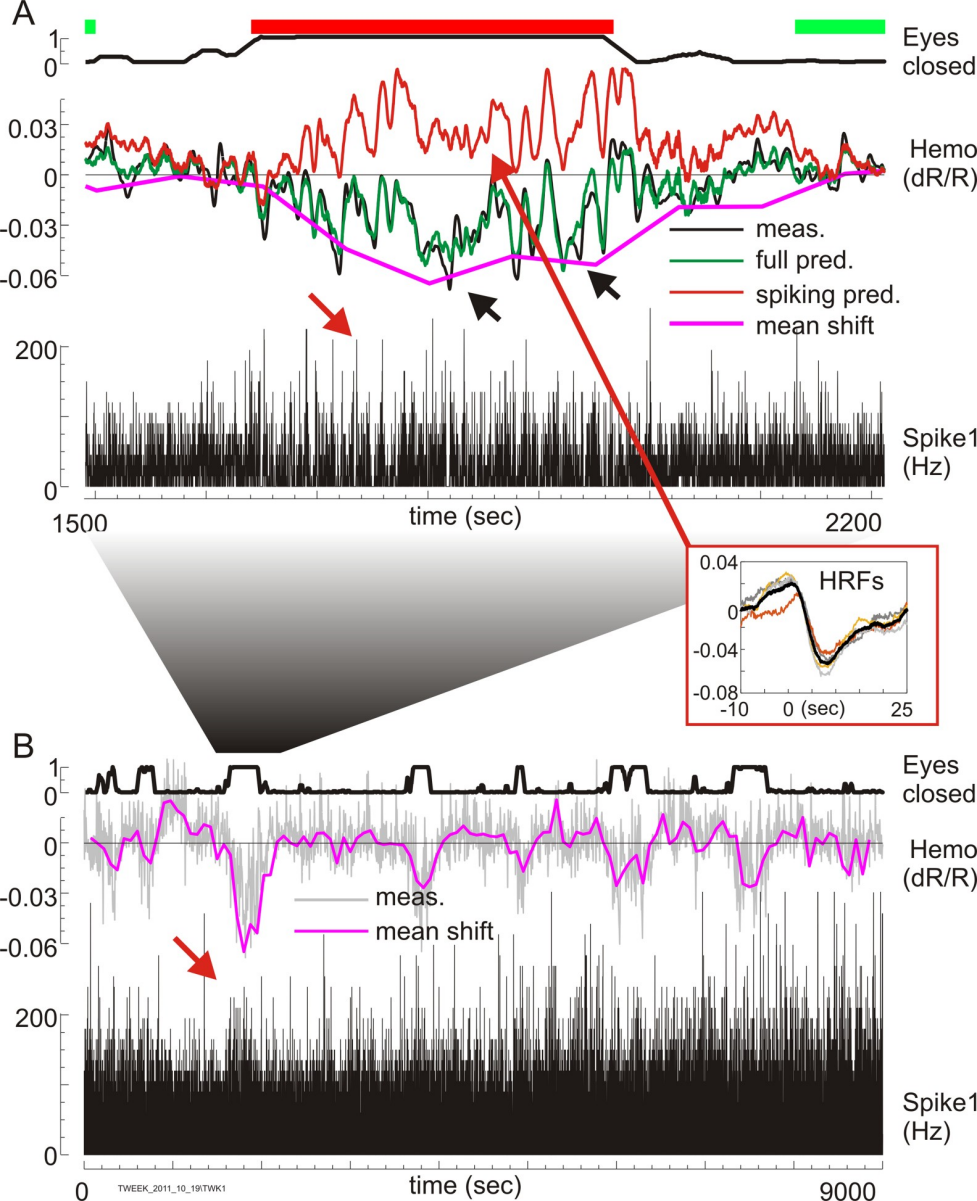
Hemodynamic response during rest is the sum of a high-frequency component predicted linearly by spiking plus an additional mean shift not predicted by spiking. The same data set as in Fig 7 is shown at a temporal resolution of 66.7 ms (15 Hz camera frame rate). (A) Expanded view of the sections of “Eyes closed,” “Hemo,” and “Spike1” data enclosed by a red box in Fig 7A, along with three alternate predictions of the measured “Hemo” response. “Eyes closed” is shown as in Fig 7A, top trace, with green and red bars indicating periods of task engagement and rest. Red arrow over the “Spike1” points to peaks of high instantaneous spike rate, indicating burst of spiking despite lower mean spike rate over this epoch (24.7 spikes/second average under “Eyes closed” red bar, Panel A, versus 29.4 spikes/second average in the two flanking green sections where eyes were open; same data as in Fig 7A, red asterisk). “Hemo” refers to 4 different hemodynamic traces color-coded as in the key. Black (“meas.”) = the measured response, same data as in Fig 7A. Green (“full pred.”) = full prediction following deconvolution. Red (“spiking pred.”) = linear prediction from spiking using deconvolved HRFs. The inset (“HRFs”) shows optimal HRF kernels from deconvolution windows in the “Eyes-closed” segment; colors are arbitrary. Magenta (“mean shift”) = fit to the intercept term in the design matrix, estimating components not predicted by spiking (see accompanying text). Black arrowheads pertain to an additional analysis in supplementary data; they point to two segments marked for comparison with an alternate deconvolution and prediction made without intercept terms in the design matrix (see S8 Fig). (B) Results of the deconvolution and prediction as in (A), shown over the full experiment. (The location of the expanded section in panel A is also indicated.) Only measured spiking, measured hemodynamic trace, and deconvolved “mean shift” are shown; full and “spiking” predictions are not shown to avoid clutter. Red arrow marks a burst of high instantaneous spiking despite lower overall mean; this burst is expanded in (A). HRF, hemodynamic response function.

Comparing hemodynamics and spiking for the same data set at the higher imaging temporal resolution (15 frames/second; Fig 8) supported our contention that the large blood volume increases at rest are not predicted from spiking. This conclusion was not immediately apparent on qualitative inspection (Fig 8A; same data segment as enclosed in the red box in Fig 7A). At the higher temporal resolution, the spiking response showed expected [60] bursts of high instantaneous spike rate (red arrow, Fig 8A) that stood out despite the overall reduction in mean spike rate as the animal rested with eyes shut (red asterisk in “Spike1,” Fig 7A). The corresponding blood volume measurements showed large swings in amplitude that appeared, qualitatively, to follow the bursts of spiking. Our earlier work showed that the recorded hemodynamics is poorly predicted by spiking when the animal is engaged in his task, because of the presence of the task-related response (see S1 Fig; [31,44,55]). But there should be no task-related response, by definition, when the animal is disengaged from the task with his eyes shut and the hemodynamics could in principle be predictable from spiking. It was thus important to test the relationship between the two at this higher temporal resolution.

We tested using deconvolution—i.e., multilinear regression (see Methods, Eqs 9–12), which has the advantage that it makes no assumptions about HRF shape [61]. The deconvolution was done over partially overlapping 150-second windows (75-second steps; 150 seconds typically covered 10 trials) to get adequate temporal resolution for tracking rest states (e.g., the rest epoch in Fig 8A, indicated by the red bar, lasts about 400 seconds. Shorter deconvolution windows led to excessive noise). Each design matrix contained not only the spiking regressor for the given deconvolution window but also additional intercept and slope terms. The intercept is analogous to the “y intercept” in 1D linear regression, quantifying an inhomogeneous addition to a homogeneous linear equation. Here, we defined it as an estimated inhomogeneous “mean shift” in the hemodynamic response, in addition to hemodynamic components that are linearly predictable from spiking. The full prediction using the deconvolved HRF kernel plus additional “mean shift” matched measured hemodynamics very well overall (Fig 8A, compare “Hemo, full pred,” green with “Hemo, meas,” black. Goodness of fit, *R*^*2*^ = 0.94, over this rest epoch). The HRFs from deconvolution windows falling within the rest epoch also matched each other well and resembled canonical HRFs (inset “HRFs,” Fig 8A; also see S5 Fig for an example canonical HRF). They predicted the high-frequency fluctuations in the measured hemodynamics well, indicating that these high-frequency terms are accounted for by spiking (Fig 8A, “Hemo, spiking pred,” red). However, they failed to account for the increase in the mean blood volume, predicting a decrease instead (prediction rising above baseline), which is consistent with the decrease in the local mean spiking. The measured increase in the blood volume was well accounted for, on the other hand, by the fitted “mean shift” (Fig 8A, “Hemo, mean shift”; the slope terms made only small contributions). The same pattern was seen over the full 2.5-hour recording (Fig 8B). Linear predictions from spiking matched the high-frequency fluctuations of hemodynamics during rest epochs whereas the additional mean shift tracked the mean measured hemodynamic response (compare Fig 8B, “mean shift” with “Hemo” trace in Fig 7A). This result supports the suggestion that the large changes in mean blood volume during rest were likely driven by a mechanism acting in addition to spiking. Similar results were obtained for all extended recording sessions, including ones in which the mean spike rate increased during rest.

It could be argued that the deconvolved “mean shift” is just the fit to the intercept term that we chose to include in the design matrix. It would thus necessarily fit the mean of the measured response, by design, with no additional physiological significance. We tested by fitting the same data, using an identical deconvolution approach but without intercept terms in the design matrix (see S8 Fig). The resulting prediction was much worse at matching the measured hemodynamics. In addition, the deconvolved HRFs obtained from this new fit were markedly different from canonical spiking HRFs in two ways. First, the HRFs now incorporated the mean CBV for their respective deconvolution windows. They thus acquired large mean shifts that made them apparently acausal with large nonzero values prior to time zero. In addition, HRFs from successive deconvolution windows were noisy and matched each other poorly. This distinctly poorer fit without the intercept term suggests that the “mean shift” in the full fit (Fig 8) represents a physiological component of the hemodynamic response during the eyes-closed, disengaged behavioral state.

## Discussion

Our goal is to understand the task-related endogenous component of hemodynamic responses recorded from visual cortex of subjects engaged in cued, predictable tasks. The existence of such responses has been known for more than a decade [16,33,35–41]. Their substantial strength relative to other brain hemodynamic components is well recognized [34]. Recent studies suggest their relevance to sensory processing [33,35]. Yet they have not been adequately studied, and little is known about their underlying mechanism or behavioral significance. Here, we consider the behavioral correlates of one particularly prominent task-related response recorded by us in V1 of behaving macaques [31], which is likely analogous to responses seen in human visual cortex [16,37].

Our work here suggests that this task-related response reflects brain mechanisms associated with the degree of task engagement. On increasing reward size to get the animal more engaged, the most notable effect, trial-by-trial, was improved temporal precision: the response became consistently more crisply aligned to task timing. It also became modestly stronger. At a slower timescale, different levels of task engagement led to consistent shifts in the mean local blood volume. High-reward blocks led to consistent decreases in the mean blood volume, whereas low-reward blocks led to corresponding increases. This effect was even more pronounced when the animal disengaged completely from his task and rested with eyes closed. The mean blood volume increased strikingly during these breaks, returning to baseline when the animal resumed working (also see [62]). Other than a high-frequency component while the animal slept, none of the hemodynamic measurements at any time scale—whether trial-by-trial or averaged across blocks while the animal worked, or the mean while the animal slept—could be accounted for by concurrent local spiking. On a methodological note, we recorded the hemodynamic response using ISOI at a wavelength tuned for measuring cortical blood volume. Such recordings have a steadier and more reliable baseline than blood oxygen level–dependent (BOLD) fMRI, suffering much less from instrumental noise and drift. This allowed us to monitor the response continuously over many hours, thus obtaining the reported results, including the prominent mean shifts.

We propose that the task-related hemodynamic response and the effects reported in this paper comprise a marker of task-specific arousal tied to the level of engagement during an extended and possibly repetitive task. The term “arousal” is used in different senses in different areas of research, from arousal during tasks such as here to arousal in the face of fear or danger to nonspecific arousal along the sleep–wake axis [48]. The literature on task-specific arousal thus suggests avoiding the term “arousal” in favor of “vigilance,” “alertness,” or “sustained attention” [48–50]. This condition of sustained engagement in a task is known to fluctuate between states of higher stability, which are less prone to error (“in the zone”), and states that are more unstable and error-prone (out of “the zone”; see, e.g., [50]). The state of being “in the zone” is marked by higher regularity and temporal precision; responses “in the zone” show less variability in reaction time, trial-by-trial, even when the mean reaction time remains unchanged overall [50]. The state is further enhanced by reward, which leads to even less variability in reaction times, in a manner that appears distinct from increased arousal [63]. This behavioral result may have a physiological analog in our finding of improved temporal precision or regularity of the task-related hemodynamic response during high reward (Fig 3 and accompanying text). An attractive possibility is that the task-related response reflects the behavioral state variously labeled “vigilance,” “alertness,” or “sustained attention” in the cited literature. Our term “task engagement” is a shorthand reflecting this possibility as well as a nod to the initial report of this hemodynamic response component, which described it as “task structure” related [16]. Much remains to be done to flesh out these connections.

An important question remains: How closely does the task-related hemodynamic response we record in macaque V1 correspond to the response identified in human visual cortex? And how distinct is it from selective visual attention [16]? Currently, the strongest evidence is that although varying substantially in time, the task-related response in the macaque is spatially homogeneous over the imaging window (a circular region 15 mm in diameter, typically extending approximately 1–6° eccentricity [32]). This makes it unlikely to be selective attention at the fovea (e.g., for the task of discriminating the fixation cue color) that should lead to spatially graded activation over this cortical extent [16,64,65]. Additional evidence comes from the response timing. If it were selective attention cued to the fixation point, its time course at fix onset should be stereotyped independent of trial length. That was not the case in an earlier test; the starting time course even switched sign when switching between blocks of short versus long trials (e.g., 8 versus 20 seconds; see Fig 3 and Supplementary Fig S9 in [31]). This result also speaks to a corollary question arising from our describing the response as being entrained to task timing. It could be argued that the hemodynamics and the sympathetic-like changes in HR and pupil are, instead, responses to the reward acting as a stimulus. However, our earlier results noted above showed the hemodynamics to be entrained to the expected timing of upcoming trials rather than a stereotyped response to the reward. It should thus be interpreted as task-entrained albeit modulated by the current reward. However, although the evidence is strongly suggestive, the questions are interesting and open and remain topics of ongoing research in the lab.

What could be the underlying mechanism or function? Although the poor prediction by recorded spiking does not rule out control by a small, hard-to-measure set of specialized neurons, it also suggests a different underlying mechanism such as neuromodulatory input (e.g., see [66]). The strong sympathetic-like responses (Fig 1) suggest the basal forebrain-cholinergic (BF-ACh) or the locus coeruleus-adrenergic (LC-NA) systems, both of which are linked to wakeful states, arousal, and attention. They powerfully facilitate hemodynamic responses via modulation of stimulus-evoked neural responses (reviewed in [67]) with additional control of cortical blood flow [68] through direct modulation of microvessels [69,70] or via astrocytes [71] or pericytes [72]. Other neuromodulators such as dopamine could also be involved [73]. All of this can lead to robust neuromodulator-mediated increases [68,74,75] or decreases [70–72,76] in cortical blood flow. Our finding of a stereotyped response shape independent of temporal precision could be accounted for by a mechanism in which the response timing is determined in a distal nucleus through temporal dynamics local to that nucleus. The result could then be transmitted in an all-or-nothing manner, like an action potential, to the target (here, V1), where it could release a fixed quantum of neurotransmitter. In addition, there could be a contribution from myogenic mechanisms independent of neural input, as suggested in a recent study of ongoing vascular fluctuations [77]. A combination of such mechanisms could modulate neural responsivity and vascular tone in a manner that reflects the level of task engagement. In single trials, this could help refresh the local blood supply ahead of task onsets [78]. Over more extended periods, it could also shift the mean vascular tone—e.g., by slow accumulation of the active substance—to higher values for high task engagement and the converse for low. Such a mean shift could account for the surprising finding of progressively lower mean blood volume for higher task engagement, since higher vascular tone does imply narrower blood vessels and thus lower tissue fraction occupied by blood. The increased vascular tone could have additional functional benefits of higher precision in stimulus-evoked hemodynamic responses. For example, adrenergic increase in vascular tone has been shown to lead to spatially and temporally sharper vascular responses to neural activity [71]. Exploring these issues through targeted experiments in behaving animals would be crucial to understanding brain mechanisms of task engagement.

## Methods

### Experimental model and subject details

Animal use procedures were in accordance with the United States NIH Guide for the Care and Use of Laboratory Animals and were approved by the IACUC of Columbia University and the New York State Psychiatric Institute (Animal Care Protocol AC-AAAU1456). Two male rhesus macaques (*Macaca mulatta*) were used in the study. Access to water was scheduled to training or recording sessions that lasted 3–5 hours per day. Eye fixation and pupil diameter were recorded using an infrared eye tracker (ISCAN [79]). Before training, each animal was implanted with a stainless steel or titanium head post. After training, craniotomies were performed over the animals’ V1, and glass-windowed stainless steel or titanium recording chambers were implanted for subsequent ISOI in the behaving animal (see section “ISOI” below). The craniotomy exposed a 20-mm diameter area of V1 covering visual eccentricities from about 1 to 10°. The exposed dura was resected and replaced with a soft, clear silicone artificial dura (GE Silicone RTV615 001). Recording chambers and artificial dura were fabricated in our laboratory following published designs [80,81]. Chambers were opened regularly for cleaning, testing for infection, and treating if necessary, following published protocols [43].

### Method details

#### Summary

Extracellular electrode recording was carried out simultaneously with ISOI from V1 of behaving monkeys performing a periodic visual fixation task. Task and recording methods are essentially identical to those in earlier papers from our lab [31,44,45,55].

#### Task and reward schedules

All experiments were based on a simple fixation task carried out either under essentially complete darkness or in the presence of visual stimuli. In both conditions, animals held fixation periodically, cued by the color of a fixation spot (fixation window: 1.0–3.5 deg. diameter; monitor distance: 133 cm; fixation duration: 3–5 seconds; trial duration: 9–22 seconds; all parameters fixed for a given experiment but variable between experiments). A juice reward followed every correct (unbroken) fixation, with no time-out or other punishment for errors. The primary behavioral manipulation consisted of systematically changing reward size.

For fixation trials in the dark room, the monitor was covered and the fixation point was presented behind a pinhole [31]. Reward sizes were alternated between high (typically 0.45 ml per correct trial, ranging from 0.35 to 0.6 ml) and low (typically 0.15 ml per correct trial, ranging from 0.1 to 0.2 ml. High- and low-reward sizes were fixed for an experiment; they were selected per day based on the animal’s willingness to work for the low reward). Rewards were alternated in blocks (typically 10 correct trials each; some experiments had longer blocks; some experiments had blocks of variable size). The animal had to correctly complete the full set of trials per block—i.e., not counting error trials—before the reward switched. Trials were grouped into “high-reward” and “low-reward” blocks for analysis. Errors were identified by the block in which they occurred—i.e., as “high” or “low” reward based on the preceding correct trial. Continuous sequences of error trials were counted as a single error to avoid arbitrary overcounting during epochs in which the animal disengaged from work and took a nap. Thus, each counted error corresponded to a break following one or more correct trials. These experiments accounted for the majority of the reported results. For visually stimulated trials (Fig 5 and S5 Fig), the animals were passively shown gratings of different contrasts while holding fixation (sine-wave gratings; contrasts doubled in steps ranging typically from 6.25% to 100%; mean luminance = background luminance = 46 cd/m^2^; spatial frequency: 2 cycles/deg; drift speed 4 deg/second; diameter 2–4 deg; orientation optimized for the electrode recording site. These data are reanalyses of earlier experiments designed to relate hemodynamics to electrophysiology over a wide dynamic range of stimulated responses [44,45,55]). Reward sizes for these experiments increased progressively from a baseline (typically 0.2 ml per correct trial) to a maximum value (typically 0.6 ml per correct trial) for each successive correct fixation to keep the animals motivated. Again, the lowest reward size per day was chosen based on the animals’ willingness to work. For analysis, trials were grouped into “high-” and “low-reward” sets relative to the median reward. Sequences of errors were also counted as single errors for these experiments, as with the dark room. However, errors were not identified as “high” or “low” reward. Since rewards were increased progressively for correct trials, errors (which often occurred at the end of a sequence of trials) typically followed a high reward; but that association was not informative.

#### ISOI

ISOI is based on the finding that in vivo and in the visible spectrum, changes in light absorption in cortical tissue primarily measure changes in oxy- and deoxyhemoglobin in the blood flowing through cortical blood vessels [51,82,83]. ISOI deduces hemodynamic responses by imaging changes in light reflection at relevant wavelengths off the exposed cortical surface. CBV and oxygenation changes measured using ISOI can be used to predict concurrently measured fMRI responses [53], making ISOI in effect an optical analog of fMRI albeit restricted to upper layers of exposed cortex. We imaged at 530 nm (green), an isosbestic wavelength that is equally absorbed by oxy- and deoxyhemoglobin. Increased absorption of light at this wavelength thus measures increased cortical tissue fraction of hemoglobin—in effect local cortical blood volume, independent of oxygenation state [54]. After the animals had recovered from surgery, we used this technique to image their V1 through the glass window of the recording chamber routinely while they engaged in the fixation task. Imaging hardware consisted of the following: camera (Dalsa 1M30P; binned to 256 × 256 pixels, 7.5 or 15 frames per second) and frame grabber (Optical PCI Bus Digital; Coreco Imaging). Imaging software was developed in our laboratory in C++ based on a previously described system [84]. Illumination was provided by high-intensity LEDs (Agilent Technologies, Purdy Technologies). The lens was a macroscope [85] of back-to-back camera lenses focused on the cortical surface. Imaging, trial data (trial onset, stimulus onset, identity and duration, etc.), and behavioral data (eye position, pupil size, timing of fixation breaks, fixation acquisitions, trial outcome) were acquired continuously. All data analyses were performed offline using custom software in MATLAB (MathWorks; RRID:nlx_153890).

#### Electrophysiology

Electrode recordings were made simultaneously with optical imaging. Recording electrodes (FHC, AlphaOmega; typical impedances approximately 600–1,000 kΩ) were advanced into the recording chamber through a silicone-covered hole in the external glass window, using a custom-made low-profile microdrive. Recording sites were mostly but not exclusively confined to upper layers. Signals were recorded and amplified using a Plexon recording system (RRID:nif-0000-10382). The electrode signal was split into spiking (100 Hz to 8 kHz bandpass) and LFP (0.7–170 Hz). Subsequently, an additional analog 2-pole 250-Hz high-pass filter was applied to spiking, effectively eliminating any spectral power overlap between LFP and spiking. No attempt was made at isolating single units, and all measured spiking was multiunit activity (MUA) defined as each negative-going crossing of a threshold = roughly 4× the r.m.s. of the baseline obtained while the animal looked at a gray screen. The LFP recording was analyzed to obtain two bandpass-limited measurements in the beta- and theta-range frequency bands (15–25 Hz and 3–7 Hz, respectively; multitaper spectral analysis using the Chronux MATLAB toolbox). This gave an LFP measure of (low) vigilance defined by the square root of the ratio of power in theta versus beta.

#### Analysis: Preprocessing

The imaging measurement was averaged over the imaged area, frame by frame (frame rate: 7.5 or 15 frames/second), and then divided by the mean value of this quantity for the given experiment (over all trials). This converted the measurement per image frame into $\frac{dR}{R}$ (i.e., the fractional change in light reflected off the cortical surface). At the particular imaging wavelength of 530 nm, the negative of this quantity ($-\frac{dR}{R}$) is proportional to the fractional increase in local tissue hemoglobin—i.e., the fractional increase in local cortical blood volume [54]. The $\frac{dR}{R}$ was then detrended, and a prominent pulse artifact was filtered out from the measured hemodynamics using Runline (Chronux) with a window of 2 seconds. This filtered $\frac{dR}{R}$ defined the measured hemodynamic response for all calculations.

The pulse artifact was used to estimate the instantaneous HR after upsampling 8× and identifying peak times and thus the local pulse rate. Both the estimated HR and the spiking measurements were then resampled and aligned to the imaging frames. Neither the imaging nor spiking nor estimated HR were further temporally filtered. Unlike in our earlier papers, we did not high-pass filter to remove slow fluctuations [31,44,45,55], specifically so as to be able to estimate fluctuations over slow timescales of many minutes.

#### Template matching (dark-room experiments: Fig 2)

The amplitude and timing of the task-related response, per trial, were estimated as the height and location of the corresponding peak of a *Template Match*. This *Template Match* consisted of the continuous, normalized dot product of a template with the measured hemodynamic response. The calculation involved the following steps:

1. The default template “*Tmplt*” was defined to be the one-trial-long mean hemodynamic recording (z-scored to give “*H*(*t*)”) aligned to trial onsets, averaged across all correct trials, and mean-subtracted.
2. This *Tmplt* was then slid over *H*(*t*) in unit time steps (at the resolution of the imaging frame rate; e.g., 66.7 ms for 15 frames/s). At every time point *t*, the *Template Match* was defined to be the local dot product over the one-trial-long section of *H* centered on *t*, normalized by the (fixed) sum of squares of the *Tmplt* (Fig 2B):
  $$Template\;Match=\frac{H(t).Tmplt}{\sum {\left|Tmplt\right|}^{2}}$$ (1)
  This expression is identical in form to the Pearson’s correlation between the *Tmplt* and the same one-trial-long segment of *H*(*t*), other than the normalization. Thus, like Pearson’s r, this expression would have local maxima where the local one-trial-long segment of *H*(*t*) matched the *Tmplt* in shape (Fig 2B). The *Template Match* is also invariant to shifts in the mean of *H*(*t*), since the *Tmplt* is mean-subtracted and thus integrates to zero over any additional constant. However, unlike Pearson’s r, this expression carries scale information. Pearson’s r has the standard deviation of both arguments in the denominator, making it scale-invariant. The *Template Match* on the other hand, with its fixed normalization independent of *H*(*t*), scales linearly with the amplitude of fluctuation in *H*(*t*). Thus, peaks of the *Template Match* carry information about both timing and amplitude of the task-related response per trial.
3. For computational efficiency in MATLAB, the above expression was rewritten as the normalized convolution of *H*(*t*) with the time-reversed version of the template *Tmplt*:
  $$Template\;Match=\frac{H(t)*TmpltTR}{\sum {\left|Tmplt\right|}^{2}}$$ (2)
  where *TmpltTR* is the time-reversed *Tmplt*; i.e., *TmpltTR*(*t*) = *Tmplt*(−*t*), and the symbol * denotes convolution. The denominator for normalization remains unchanged. This expression translated to the following script using MATLAB functions *conv* and *sum*:
  $$Template\;Match=\frac{conv(H,TmpltTR{,}^{\prime }same^{\prime })}{sum({\left|Tmplt\right|}^{2})}$$ (3)
  Peaks of *Template Match* were then identified as zero crossings of the first derivative at points where the second derivative was negative (marked by red dots in Fig 2B).
4. Alternate template matches used the same formalism but with different definitions of *Tmplt*. Thus, the *Tmplt* in S2 Fig was defined as the one-trial-long mean *H*(*t*) aligned to a point that was one-quarter trial ahead of trial onsets, averaged across all correct trials, and mean-subtracted. All other steps were the same.

#### Template matching (with visual stimuli present: Fig 5, S5 Fig)

This involved two separate sets of steps.

1. Estimating the task-related response from the net recorded hemodynamic response:
  - We modeled the net recorded response as a linear sum of stimulus-evoked and task-related components. The stimulus-evoked response was modeled as the convolution of concurrent spiking with an “*HRF*” kernel. The task-related component was estimated iteratively. Our earlier approach [55] had modeled it as a stereotyped task-related function (“*TRF*”) that was identical in timing and amplitude for each correct trial. Here, however, we specifically need to estimate trial-by-trial variations in response timing and amplitude. As a first step, we assumed that the *TRF* had a fixed shape that could be estimated from the mean across trials. Optimal mean *HRF* and *TRF* kernels were obtained by fitting the mean recorded responses, separated by contrast, with the following equation, identical to Eq 1 in [55]:
    H(t)=HRF*S(t)+TRF*Trl(t) (4)
    *H*(*t*) is the recorded hemodynamics and *S*(*t*) the concurrently measured spiking, and HRF*S(*t*) models the stimulus-evoked response. The second term on the RHS models the task-related response as a *TRF* kernel convolved with the set of delta functions at trial onsets, "*Trl*(*t*)”. The symbol * denotes convolution over time.
    The *HRF* kernel was parametrized, as before [31,44,45], as a gamma-variate function of time *t*:
    $$HRF(t,\tau ,W,A)=A{\left(\frac{t}{\tau }\right)}^{\alpha }\exp\left(-\alpha \frac{t-\tau }{\tau }\right)$$ (5)
    The *HRF* parameters fitted during optimization are the amplitude *A*, time to peak *τ*, and full width at half maximum *W* [31,86,87]. The factor $\alpha =8.0\times log(2.0)\times {\left(\frac{\tau }{W}\right)}^{2}$.
    The *TRF* kernel was parametrized as the finite sum of a Fourier time series:
    $$TRF(t,a,b,P,N)=\sum_{n=1}^{N}\left(a_{n}\;\cos\left(n\frac{2\pi }{PT}t\right)+b_{n}\;\sin\left(n\frac{2\pi }{PT}t\right)\right)$$ (6)
    Although the Fourier series was based on the trial period *T*, the fundamental Fourier period was allowed to vary as a fraction *P* of the trial period and optimized in the fit. The parameters *a*_*n*_ and *b*_*n*_, (with *n* ranging from 1 to the total number of terms in the Fourier series, *N*) are the pairs of cosine and sine coefficients, respectively, for the *n*th Fourier term. We showed earlier that only the fundamental and first harmonic—i.e., *N* = 2, carry significant information [55]. Thus, there are eight parameters in the model: three for the *HRF*, the two pairs of *a*_*n*_ and *b*_*n*_, and *P*.
  - All parameters were optimized simultaneously by matching the predicted to the measured hemodynamics using a downhill simplex algorithm (*fminsearch*, MATLAB methods as in [45]). To keep contrast information, we made concatenated sequences of the mean response per distinct contrast, randomized per contrast (same random sequence for hemodynamics, and spiking), and over multiple blocks (an arbitrarily large number 52, about 100× larger than a single *HRF* kernel convolution length, to minimize edge effects; we only matched traces two convolution lengths in from the edge). The error to be minimized was defined as the normalized sum squared error $\left(\frac{SS_{error}}{SS_{total}}\right)$ calculated separately per contrast and then averaged over all contrasts including the blank. This was intended to give equal weight to the fractional error at each stimulus contrast. The goodness of fit *R*^*2*^ for the optimal prediction was defined as the coefficient of determination $\left(1-\frac{SS_{error}}{SS_{total}}\right)$ calculated separately per contrast and averaged across contrasts. This, again, was intended to give equal weight to errors at each contrast. In order to reduce the chances of getting caught in a local minimum, we started with large sets of initial parameter values, independently covering an order of magnitude for each fitted parameter. The fits were robust and converged to the same optimal parameters from multiple starting values, giving us confidence that we had reached global and not local minima.
  - Next, we used the optimal fitted *HRF* thus obtained from the mean hemodynamics and spiking, averaged per contrast, to get a continuous estimate of the exogenous, visually evoked component of measured hemodynamics. This was done by convolving the optimal *HRF* with the measured spiking, including both the spiking from the controlled visual stimuli and from uncontrolled visual stimulation as the animal looked around in between fixations:
    $$H_{STIMULATED}(t)=HRF*S(t)$$ (7)
  - This estimate of *H*_*STIMULATED*_ was subtracted from the full measured hemodynamics to get an estimate of the endogenous, task-related component of hemodynamics as the residual not accounted for by spiking:
    $$H_{TASK-RELATED}(t)={H(t)-H}_{STIMULATED}(t)$$ (8)
2. Estimating task-related response peaks and amplitudes:
  - The estimate of the task-related response *H*_*TASK*−*RELATED*_(*t*) as defined above was then used, exactly like the full hemodynamic response in the dark room, to estimate response times and amplitudes trial-by-trial. As a template, we used the optimal *TRF* obtained above by fitting to mean responses. The steps for template matching and identifying and analyzing peaks were identical to those outlined in Eqs 1–3, with the *H*(*t*) being replaced with *H*_*TASK*−*RELATED*_(*t*) and the *Tmplt*(*t*) being replaced with the optimal *TRF*. Peak times and amplitudes, per trial, were obtained exactly as in the dark-room template match.

#### Tracking measured variables as animal switches from task-engaged to disengaged with eyes closed (Fig 7)

To track slow changes in all measured variables, we downsampled the data. Data were averaged using a 15-second box car that corresponded roughly to a single trial and then decimated 4×, giving in effect a smoothed 60-second sample rate. Along with MUA spike rates at individual electrodes and the measured hemodynamics, the following measurements were thus tracked:

1. “Eyes closed”: Fraction of time over the 60-second averaging window that eyes are closed. Eye closure was monitored using the output from the IR eye tracker. All blinks or eye closures appeared as sequences of missing points or “rails” (saturated output). Spontaneous eye blinks in macaques last roughly 200 ms (see [88,89]). Our own data showed a bimodal distribution with blink durations peaking either at 200 ms or multiple seconds to minutes. Thus, sequences of missing points lasting <500 ms were considered regular spontaneous blinks while awake and were marked as having duration = 0. Sequences lasting >500 ms were categorized as eye closures, and their durations were included in the moving average.
2. “LFP measure”: square root of the ratio of spectral band–limited power in the theta (3–7 Hz) and beta (15–25 Hz) frequency bands, each normalized by its standard deviation over the entire experiment. We chose this particular ratio to get a measure that was high during epochs of low engagement in the task to match “Eyes closed,” since theta power increases sharply on transitions from high to low engagement or to sleep [48]. We took the square root of the ratio to compress the measure to approximately 0–1 to make it comparable to “Eyes closed.” There was no attempt to separate the resting state more finely into sleep stages, since the goal was a broad separation into states of “task-engaged” versus “resting” with a time resolution of 60 seconds.
3. “Spike1,” “Spike2”: MUA responses recorded from two electrodes spaced 1 mm apart, in imaged V1.
4. “Spk1-Spk2 Corr”: Pairwise correlation of MUA spike rate from the two electrodes, calculated over a 1-second moving window.
5. “HR”: Obtained from the pulse artifact in the measured hemodynamics, after upsampling 8× and identifying peak times and thus the local pulse rate. This instantaneous pulse rate, estimated at pulse time points, was then interpolated with spline smoothing to the imaging time base (7.5-Hz or 15-Hz sample rate depending on the experiment).

#### Deconvolution, i.e., multilinear regression (Fig 8)

We started with the assumption that the measured hemodynamics *H*(*t*) can be predicted from local spiking *S*(*t*) using a homogeneous linear equation, along with two inhomogeneous terms: a scalar *Intercept* and a linear *Slope* in each 150-second window, at the resolution of the camera frame rate:

H(t)=HRF*S(t)+Intercept+Slope (9)

There are no assumptions about the shape of the *HRF* other than that it does not extend more than 10 seconds prior to time 0 and is back to baseline about 25 seconds after time 0. Using the formalism of deconvolution, this expression can be rewritten as a matrix equation

H=S×HRF+Intercept+Slope (10)

where *H* is a column vector of recorded hemodynamic responses (at the temporal resolution of the camera frame rate, 15 Hz); *S* is the spiking regressor expanded into a stimulus convolution matrix (SCM) [37,61]; the symbol × indicates matrix multiplication; and *HRF*, *Intercept*, and *Slope* here refer to the same terms as in Eq 10 but expressed as column vectors. The SCM was constructed as a Toeplitz matrix comprising a horizontal concatenation of spiking column vectors, with circular time shifts ranging from −10 seconds to +25 seconds relative to t = 0. Formally, the SCM *S* can be extended (“*S*_*e*_”) to incorporate the *Intercept* and *Slope* by horizontally concatenating the two additional column vectors: a column of ones for the *Intercept* and a linear ramp from −1 to 1 for the *Slope*. The corresponding *HRF* can be formally extended (“*HRF*_*e*_”) by two coefficients, one for the *Intercept* and another for the *Slope*.

$$H=S_{e}\times {HRF}_{e}$$ (11)

Assuming that any noise is Gaussian and has zero mean, the optimal deconvolved *HRF*_*e*_ can then be estimated using a least-squares solution to the linear regression [37,61]:

$${HRF}_{e}={\left({S_{e}}^{T}\times S_{e}\right)}^{-1}\times {S_{e}}^{T}\times H$$ (12)

where the superscript ^*T*^ indicates the matrix transpose, ^-1^ indicates the matrix inverse, and × indicates matrix multiplication. The full prediction using the optimal deconvolved *HRF*_*e*_ kernel is then computed using Eq 11. Similarly, just the (linear, homogeneous) prediction from spiking is obtained by taking the matrix multiplication over all column vectors of *S*_*e*_, save the last two, with all coefficients of the deconvolved *HRF*_*e*_, save the last two. Conversely, just the *Intercept* term or just the *Slope* term are obtained by appropriately multiplying the last two column vectors of *S*_*e*_ with the corresponding last two coefficients of the deconvolved *HRF*_*e*_. The optimal deconvolved *Intercept* is defined as the additional “mean shift” not predicted by spiking. As with the model-based approach to fitting used earlier (see Eqs 4–6), the goodness of fit *R*^*2*^ for the optimal prediction was defined as the coefficient of determination $\left(1-\frac{SS_{error}}{SS_{total}}\right)$. This was used to compare fits made with versus without including an intercept term in the design matrix (Fig 8A versus S8 Fig).

#### Getting bootstrap estimates for significance (*p*-values)

All comparisons between distributions of amplitudes or peak times were tested for significance by bootstrapping, typically using 10,000 resamples with replacement. In cases with different numbers of trials for high and low reward, the smaller number of trials was chosen to make the bootstrap comparison. For comparing response amplitudes (e.g., Fig 3B), we tested for the median of high-reward amplitudes being less than that for low-reward amplitudes over the set of all resamples, against the null hypothesis that this difference has zero mean. We also tested for the complement—i.e., that median of low-reward amplitudes is less than that of high-reward amplitudes. For comparing widths of peak time distributions (e.g., Fig 3A), we first calculated the 2 standard deviation width (specifically, the +/− 34th percentile around the median, given the non-normal distribution) of each bootstrapped set of peak times separately for high and low reward. We then tested for 2 standard deviation for high reward being less than that for low reward over the set of all resamples, against the null hypothesis that the difference has zero mean. We also tested for the complement—i.e., that 2 standard deviations for low reward was less than that for high reward.

#### Fitting spiking to dark-room hemodynamic response using gamma-variate *HRF* (S1 Fig)

To link to spiking, the dark-room response was modeled as a homogeneous prediction from spiking, fitted by optimizing a gamma-variate *HRF* kernel using *fminsearch* as described above:

H(t)=HRF*S(t) (13)

The fitting was done separately for the high-reward and low-reward trials at each recording site. Stimulus-evoked responses were fitted using a model with a task-related component: Eqs 4–6. In each case, the optimal fitted *HRF* kernel was then convolved with the continuous recorded spiking response to give a continuous prediction. Since the spiking response included both high-reward and low-reward segments, the prediction included sections of “same” prediction (e.g., low-reward spiking convolved with the low-reward kernel) and sections of cross predictions (e.g., high-reward spiking convolved with the low-reward kernel).

## Supporting information

S1 Fig Local spiking, although appearing to predict mean hemodynamic responses in individual recording conditions, is a poor and unreliable predictor of task-related responses overall. (a,b) Mean measured responses and optimal predictions for low-reward and high-reward trials, respectively, of a data set recorded in the dark-room task. In each case, the lower panel shows the mean measured spiking; the upper panel shows the mean measured hemodynamics as well as the prediction from spiking using the corresponding optimal fitted gamma-variate HRF kernel (see color code in each column). Low reward (*N* = 148 trials), *R*^2^ = 0.73 for the optimal prediction. High reward (*N* = 140 trials), *R*^2^ = 0.42 for the optimal prediction. (c) A separate set of visually stimulated trials at the same recording site, using visual stimuli consisting of optimally oriented drifting gratings at different contrasts, as indicated by the grayscale coding (orange bar below depicts the visual stimulation period). Again, the top panel shows mean measured hemodynamics and optimal predictions grouped by stimulus contrast; predictions are shifted to the right for visibility (*N* = 141 trials total, i.e., 47 trials / contrast. *R*^2^ = 0.95). (d) The optimal fitted gamma-variate HRF kernels for the three recording conditions, color coded as shown. Note how poorly they match each other. (e) Comparing the measured low-reward hemodynamics to predictions using the low-reward dark-room set of spiking responses (as in panel a)—but with different optimal HRF kernels—from low-reward, high-reward, and stimulus-evoked sets. The cross predictions are poor (*R*^2^ of prediction using high-reward HRF = −0.014; stimulated HRF = −0.011). (f) Optimal HRFs from the full set of dark-room experiments, normalized in each case to the amplitude of the corresponding visually stimulated HRF (*N* = 56: pairs of high- and low-reward HRFs for each of 28 sets with electrode recordings). Scale truncates some HRFs of high absolute amplitude to help visualize those of smaller amplitude. Colors are arbitrary (MATLAB default). The different optimal HRFs match each other poorly, with some even reversed in sign. This makes cross prediction meaningless and suggests that apparently good predictions of mean responses in individual experiments are fortuitous. HRF, hemodynamic response function.
(TIF)

S2 Fig Increase in temporal precision with reward size is not sensitive to the choice of template used to estimate response time and amplitude. (a-d) Same example data set as in Figs 2 and 3. (a) Orange indicates the alternate template defined as the mean hemodynamic response across correct trials, aligned to a time point one-quarter cycle ahead of trial onset (i.e., starting at the dashed vertical line 4.1 seconds ahead of time 0. Single trials are shown in gray). Green background (time points 0–16.4 second) marks the timing of the earlier template for comparison (see Fig 2B, “Tmplt”). (b) New template match (orange, “Tmplt Match,” upper row) illustrated using the same segment of recorded hemodynamics (“Hemo”) as in Fig 2B. The earlier template match from Fig 2B is shown alongside for comparison (green, dashed line). Black dots identify the peaks of the new Template Match, marking locations where the “Hemo” is locally best phase-matched to the new template (see “Match Peak,” compared to “Match Trough”). (c) Distributions of response times, defined as the positions of the new template match peaks. Compare with Fig 3A (same conventions). (d) Distributions of response amplitudes using the new template match. Compare with Fig 3B (same conventions). (e, f) New response timing distribution 2 standard deviation widths and amplitude medians for high- versus low-reward trials across all experiments, including *p*-values from Wilcoxon signed rank test for the pairwise comparisons. Compare with Fig 3C and 3D (data in S24 Data).
(TIF)

S3 Fig Response timing does not correlate with fixation onset. (a) Simulation of the null hypothesis. The task-related response has a stereotyped time course following the onset of fixation. Response times would then have a constant delay following fix onset, leading to a linear relation between the two with unity slope (the delay was taken to be 10 seconds for this simulation). The observed tighter clustering of response times for high reward could result from a corresponding clustering of fixation onsets (consider projection of red dots versus blue dots on the Response Time axis). (b) Relationship between measured response time (estimated as usual with a template match) and fixation onset in an early recording session. Animals tended to hold fixation for extended periods prior to trial onset, even across multiple trials. (c) Relationship between response time and fix onset in a late recording session. Animals tended to move their eyes a lot during intertrial intervals, fixating shortly before trial onset. For both cases (b) and (c), response times were independent of fixation onset and very different from the pattern expected for the null hypothesis. In both data sets, response times for high-reward trials showed visibly lower scatter independent of fix onset (data in S25 Data).
(TIF)

S4 Fig High reward does not correlate with tighter eye movements. (a1) Mean radial eye movement per intertrial interval in an early recording session. Each dot represents a single trial (mean eye movement during 7-second intertrial intervals per 9-second trial). Horizontal lines indicate median eye movement per block of high or low reward (blocks with varying numbers [13–31] of correct trials each). Intertrial eye movements were higher in low-reward blocks. (a2) Histogram of mean eye movement per trial. (a3) Relationship between response time and eye movement per trial, colored by reward size. (b1) Mean radial eye movement in a later recording session (12-second intertrial intervals in 16-second trials; alternating blocks of 10 correct trials each; all other conventions as in panel A1). Eye movements were higher in high-reward blocks. (b2) Corresponding histogram of mean eye movements per trial. (b3) Response time versus eye movement per trial colored by reward size. Low reward leads to wider scatter of response times in both panels (a3) and (b3) despite opposite effects on intertrial eye movement (data in S26 Data).
(TIF)

S5 Fig Estimating task-related response and its template match in the presence of visual stimulation (one example data set). (a-c) Estimating optimal fitted parameters (see Methods, Eqs 4–6). (a) The mean hemodynamic response per stimulus contrast (see key), averaged across trials. The response is modeled as the sum of the stimulus-evoked component (b) and the task-related component (c). The stimulus-evoked component is modeled as the convolution (⊗) of the measured spiking with a gamma-variate HRF kernel (inset). The mean task-related component is modeled as the convolution of delta functions at trial onset with a “Mean TRF” kernel comprising a partial Fourier sum with its fundamental at the trial period (inset). Earlier work showed that the fundamental and the first harmonic terms of the Fourier series are adequate. Insets show the optimal fitted gamma-variate HRF (in b) and optimal mean TRF (in c), respectively. (d) Set of traces illustrating the process of estimating the residual task-related response and then estimating its timing and amplitude per trial by matching to a template (see Methods, Eqs 7 and 8). “Spiking,” “Hemo”: full measured responses, individually z-scored. “Hemo (predicted from spiking)” is the convolution of the spiking response with the optimal fitted HRF (b, inset). Subtracting this from the measured hemodynamic response gives the residual “Hemo (Unpredicted by spiking),” which we defined to be the task-related response. The moving-window dot product of this residual with the template (the optimal fitted mean TRF [c), inset]) gives the “Template Match” (shifted up for visibility). Timing and amplitude of task-related responses, per trial, are defined to be the location and height of each Template Match peak, as for the dark-room task. Showing a section of the full experiment of 483 trials (122 correct). (e) Set of all residual task-related responses, converted back from z-scored values, separated into trials grouped by reward size. The same data are shown in Fig 5A. HRF, hemodynamic response function; TRF, task-related function.
(TIF)

S6 Fig Comparing regression lines through alternating blocks of high and low reward, before (top panel) and after (bottom panel) removing error trials. Color coding for high (red) and low reward (cyan) is the same as in the main text. Error trials are indicated in lighter colors and are grouped with the reward block corresponding to the immediately preceding correct trial. Straight lines show regression fits. Letters (“A,” “B,” “C”) and arrows identify corresponding blocks. Blocks A and B contain individual or short stretches of error trials. C includes a roughly 400-second stretch during which the animal napped. The time axis has the same scale for both top and bottom panels, with time 0 indicating the start of the experiment; the bottom concatenates time points for correct trials. Six consecutive blocks are shown from an experiment comprising 47 blocks (482 correct trials of 684 total).
(TIF)

S7 Fig Ramp-like drifts in local blood volume are not accounted for by slow changes in local spiking. (a, c) Hemodynamics and spiking, respectively, showing correct trials from alternating blocks of high and low reward. Lines show regression fits per block (same data set as Figs 2 and 3). (b, d) Histograms with slopes of regression fits from (a), (c). (e) Simplified simulation of slow mean hemodynamic responses: triangle wave of matching period, with slopes equal to the median (absolute) slopes of the regression lines in (a) (= 4.1 × 10^−5^/second). (f) Simulated spiking response that generates the model hemodynamic response in (e) on convolving with the visually stimulated HRF for this recording site (see “HRF kernels,” S1 Fig; also, Methods). Measured spiking regression slopes (d) are only about 4× weaker than those in the simulation; but they do not alternate in sign with reward size. (g) Distributions of the ratios of measured spiking regression slope per block to the slope of the corresponding simulation, as in (e), (f), across all experiments (*N* = 752 blocks of 10 trials each, 376 blocks/reward size; from *N* = 11 experiments with electrode recordings and at least 10 blocks per reward size). *p*-Values test for the probability of the distributions being centered on zero (bootstrap, 10,000 resamples) (data in S27 Data). HRF, hemodynamic response function.
(TIF)

S8 Fig Deconvolution fit of the same data segment as in Fig 8A but with no intercept term in the design matrix. The full prediction here matches the measured response reasonably well except for a few locations with large mismatches (black arrowheads; compare with the same locations in Fig 8A). The overall goodness of fit *R*^*2*^ = 0.76, averaged over this rest epoch, is worse than for the fit with an intercept (*R*^*2*^ = 0.94; see Fig 8A, text). The inset shows HRFs from the deconvolution windows covering this rest epoch, as in Fig 8A; colors identify corresponding HRFs for the two fits. HRF, hemodynamic response function.
(TIF)

S1 Data Data for “Eye Pos” traces in Fig 1B. (XLSX)

S2 Data Data for “Hi–Lo” reward pupil dilation histograms in Fig 1B. (XLSX)

S3 Data Data for pupil traces in Fig 1B. (XLSX)

S4 Data Data for Fig 1C. (XLSX)

S5 Data Data for Fig 1D. (XLSX)

S6 Data Data for Fig 1E. (XLSX)

S7 Data Data for Fig 2A. (XLSX)

S8 Data Data for Fig 2C. (XLSX)

S9 Data Data for Fig 2D. (XLSX)

S10 Data Data for Fig 2E and 2F. (XLSX)

S11 Data Data for Fig 3. (XLSX)

S12 Data Data for Fig 4A. (XLSX)

S13 Data Data for Fig 4B. (XLSX)

S14 Data Data for Fig 4C–4F. (XLSX)

S15 Data Data for Fig 5A. (XLSX)

S16 Data Data for Fig 5B. (XLSX)

S17 Data Data for Fig 5E. (XLSX)

S18 Data Data for Fig 5F. (XLSX)

S19 Data Data for Fig 5C and 5D. (XLSX)

S20 Data Data for Fig 6C. (XLSX)

S21 Data Data for Fig 6A and 6B. (XLSX)

S22 Data Data for Fig 7B. (XLSX)

S23 Data Data for Fig 7C. (XLSX)

S24 Data Data for S2 Fig. (XLSX)

S25 Data Data for S3 Fig. (XLSX)

S26 Data Data for S4 Fig. (XLSX)

S27 Data Data for S7 Fig. (XLSX)

## Abbreviations

BF-ACh: basal forebrain-cholinergic
BOLD: blood oxygen level–dependent
CBV: cerebral blood volume
EEG: electroencephalographic
fMRI: functional magnetic resonance imaging
HR: heart rate
HRF: hemodynamic response function
IACUC: Institutional Animal Care and Use Committees
ISOI: intrinsic-signal optical imaging
LC-NA: locus coeruleus-adrenergic
LFP: local field potential
NIH: National Institutes of Health
NS: not significant
