# Supplementary figures and images for: Task-related hemodynamic responses are modulated by reward and task engagement
Source: PLoS Biol. 2019 Apr 19;17(4):e3000080. doi: 10.1371/journal.pbio.3000080 (PMC6493772; doi:10.1371/journal.pbio.3000080)

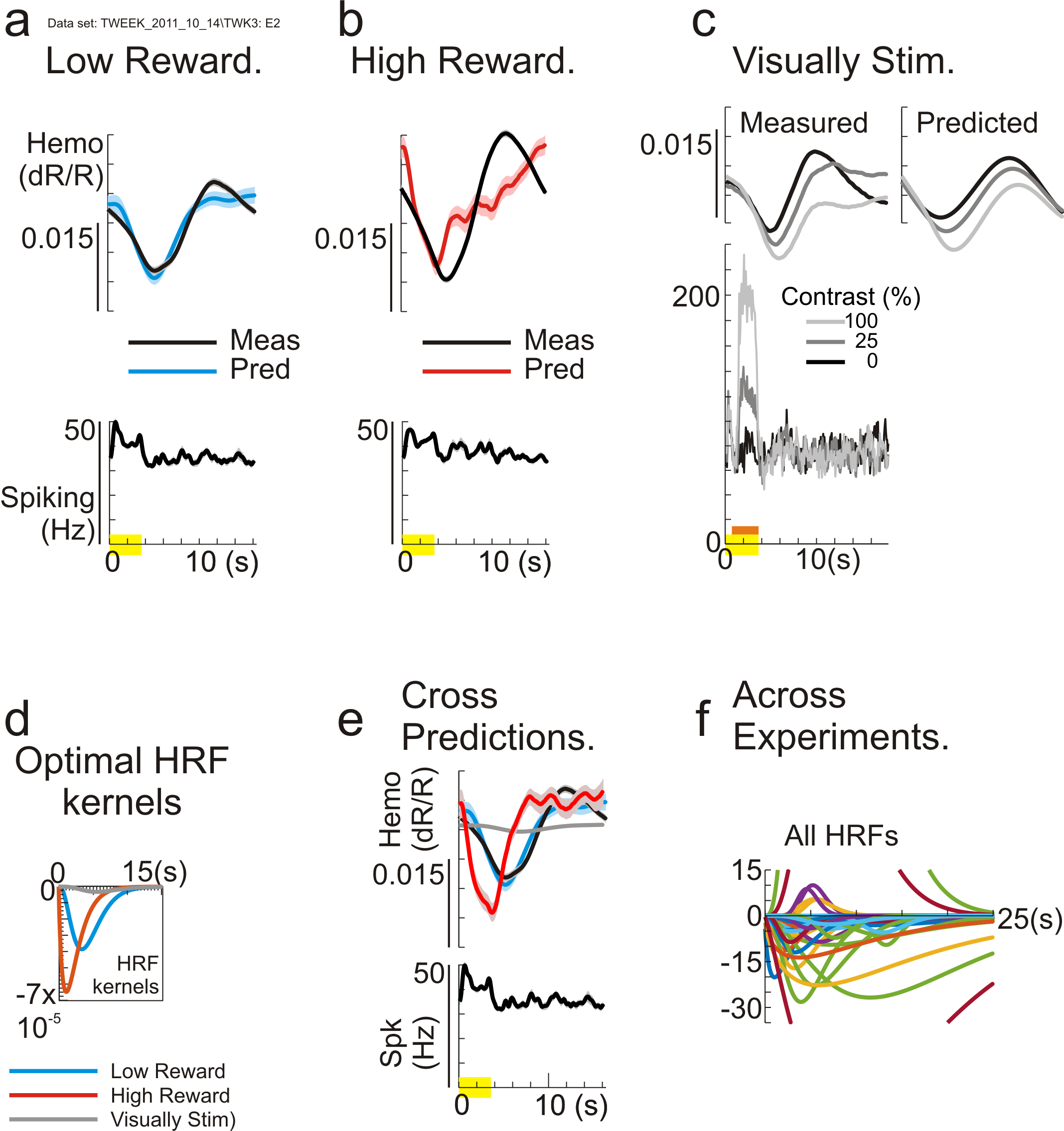

Supplement: S1 Fig — (a,b) Mean measured responses and optimal predictions for low-reward and high-reward trials, respectively, of a data set recorded in the dark-room task. In each case, the lower panel shows the mean measured spiking; the upper panel shows the mean measured hemodynamics as well as the prediction from spiking using the corresponding optimal fitted gamma-variate HRF kernel (see color code in each column). Low reward (N = 148 trials), R2 = 0.73 for the optimal prediction. High reward (N = 140 trials), R2 = 0.42 for the optimal prediction. (c) A separate set of visually stimulated trials at the same recording site, using visual stimuli consisting of optimally oriented drifting gratings at different contrasts, as indicated by the grayscale coding (orange bar below depicts the visual stimulation period). Again, the top panel shows mean measured hemodynamics and optimal predictions grouped by stimulus contrast; predictions are shifted to the right for visibility (N = 141 trials total, i.e., 47 trials / contrast. R2 = 0.95). (d) The optimal fitted gamma-variate HRF kernels for the three recording conditions, color coded as shown. Note how poorly they match each other. (e) Comparing the measured low-reward hemodynamics to predictions using the low-reward dark-room set of spiking responses (as in panel a)—but with different optimal HRF kernels—from low-reward, high-reward, and stimulus-evoked sets. The cross predictions are poor (R2 of prediction using high-reward HRF = −0.014; stimulated HRF = −0.011). (f) Optimal HRFs from the full set of dark-room experiments, normalized in each case to the amplitude of the corresponding visually stimulated HRF (N = 56: pairs of high- and low-reward HRFs for each of 28 sets with electrode recordings). Scale truncates some HRFs of high absolute amplitude to help visualize those of smaller amplitude. Colors are arbitrary (MATLAB default). The different optimal HRFs match each other poorly, with some even reversed in sign. This makes cross pred [file pbio.3000080.s001.tif]

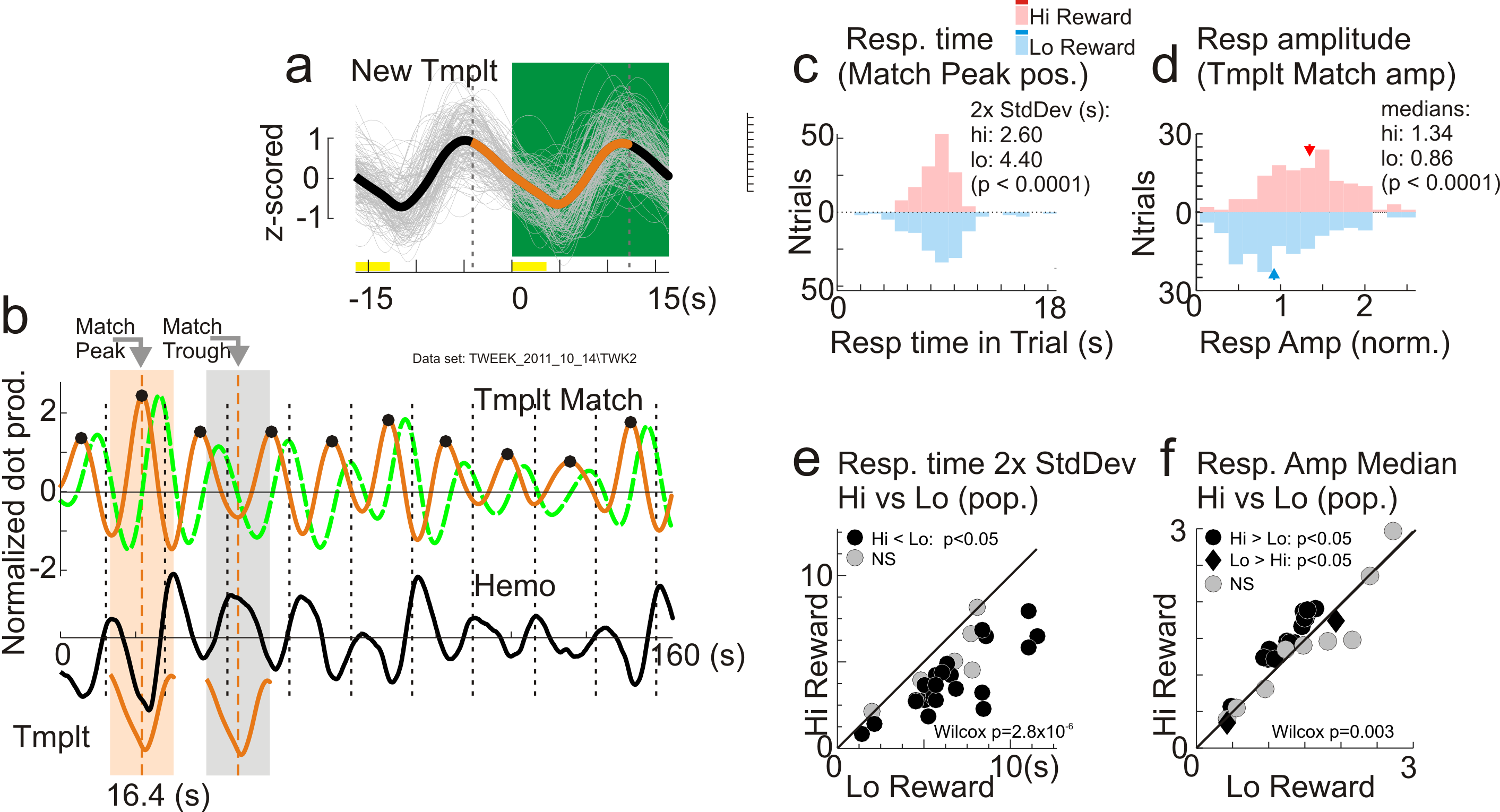

Supplement: S2 Fig — (a-d) Same example data set as in Figs 2 and 3. (a) Orange indicates the alternate template defined as the mean hemodynamic response across correct trials, aligned to a time point one-quarter cycle ahead of trial onset (i.e., starting at the dashed vertical line 4.1 seconds ahead of time 0. Single trials are shown in gray). Green background (time points 0–16.4 second) marks the timing of the earlier template for comparison (see Fig 2B, “Tmplt”). (b) New template match (orange, “Tmplt Match,” upper row) illustrated using the same segment of recorded hemodynamics (“Hemo”) as in Fig 2B. The earlier template match from Fig 2B is shown alongside for comparison (green, dashed line). Black dots identify the peaks of the new Template Match, marking locations where the “Hemo” is locally best phase-matched to the new template (see “Match Peak,” compared to “Match Trough”). (c) Distributions of response times, defined as the positions of the new template match peaks. Compare with Fig 3A (same conventions). (d) Distributions of response amplitudes using the new template match. Compare with Fig 3B (same conventions). (e, f) New response timing distribution 2 standard deviation widths and amplitude medians for high- versus low-reward trials across all experiments, including p-values from Wilcoxon signed rank test for the pairwise comparisons. Compare with Fig 3C and 3D (data in S24 Data). (TIF) [file pbio.3000080.s002.tif]

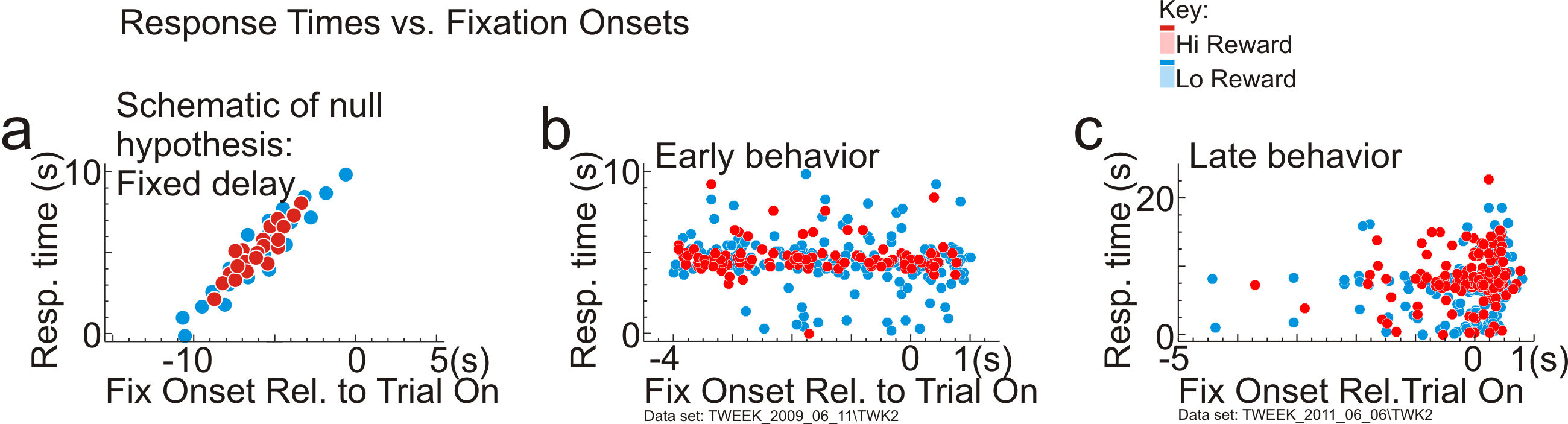

Supplement: S3 Fig — (a) Simulation of the null hypothesis. The task-related response has a stereotyped time course following the onset of fixation. Response times would then have a constant delay following fix onset, leading to a linear relation between the two with unity slope (the delay was taken to be 10 seconds for this simulation). The observed tighter clustering of response times for high reward could result from a corresponding clustering of fixation onsets (consider projection of red dots versus blue dots on the Response Time axis). (b) Relationship between measured response time (estimated as usual with a template match) and fixation onset in an early recording session. Animals tended to hold fixation for extended periods prior to trial onset, even across multiple trials. (c) Relationship between response time and fix onset in a late recording session. Animals tended to move their eyes a lot during intertrial intervals, fixating shortly before trial onset. For both cases (b) and (c), response times were independent of fixation onset and very different from the pattern expected for the null hypothesis. In both data sets, response times for high-reward trials showed visibly lower scatter independent of fix onset (data in S25 Data). (TIF) [file pbio.3000080.s003.tif]

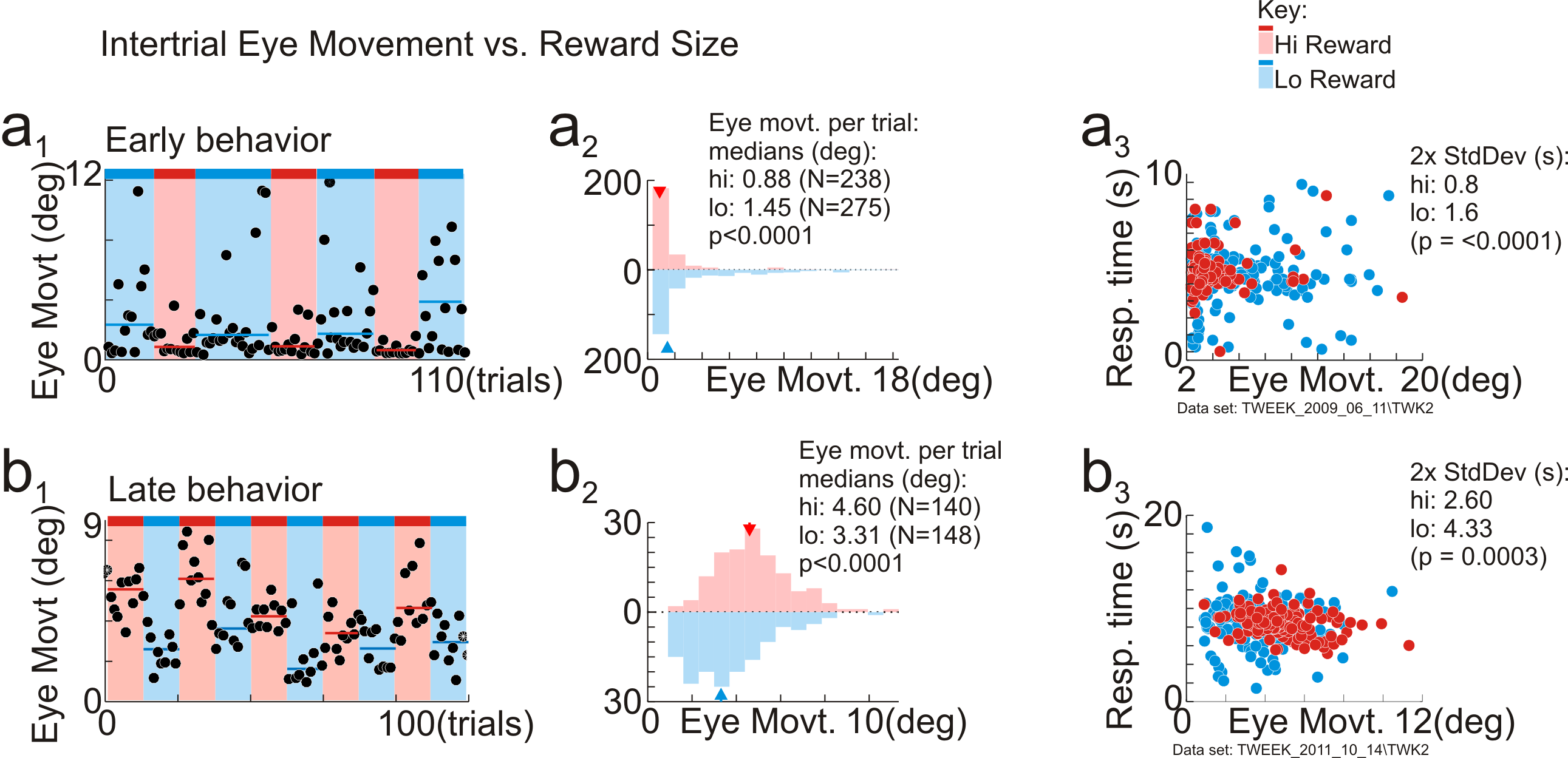

Supplement: S4 Fig — (a1) Mean radial eye movement per intertrial interval in an early recording session. Each dot represents a single trial (mean eye movement during 7-second intertrial intervals per 9-second trial). Horizontal lines indicate median eye movement per block of high or low reward (blocks with varying numbers [13–31] of correct trials each). Intertrial eye movements were higher in low-reward blocks. (a2) Histogram of mean eye movement per trial. (a3) Relationship between response time and eye movement per trial, colored by reward size. (b1) Mean radial eye movement in a later recording session (12-second intertrial intervals in 16-second trials; alternating blocks of 10 correct trials each; all other conventions as in panel A1). Eye movements were higher in high-reward blocks. (b2) Corresponding histogram of mean eye movements per trial. (b3) Response time versus eye movement per trial colored by reward size. Low reward leads to wider scatter of response times in both panels (a3) and (b3) despite opposite effects on intertrial eye movement (data in S26 Data). (TIF) [file pbio.3000080.s004.tif]

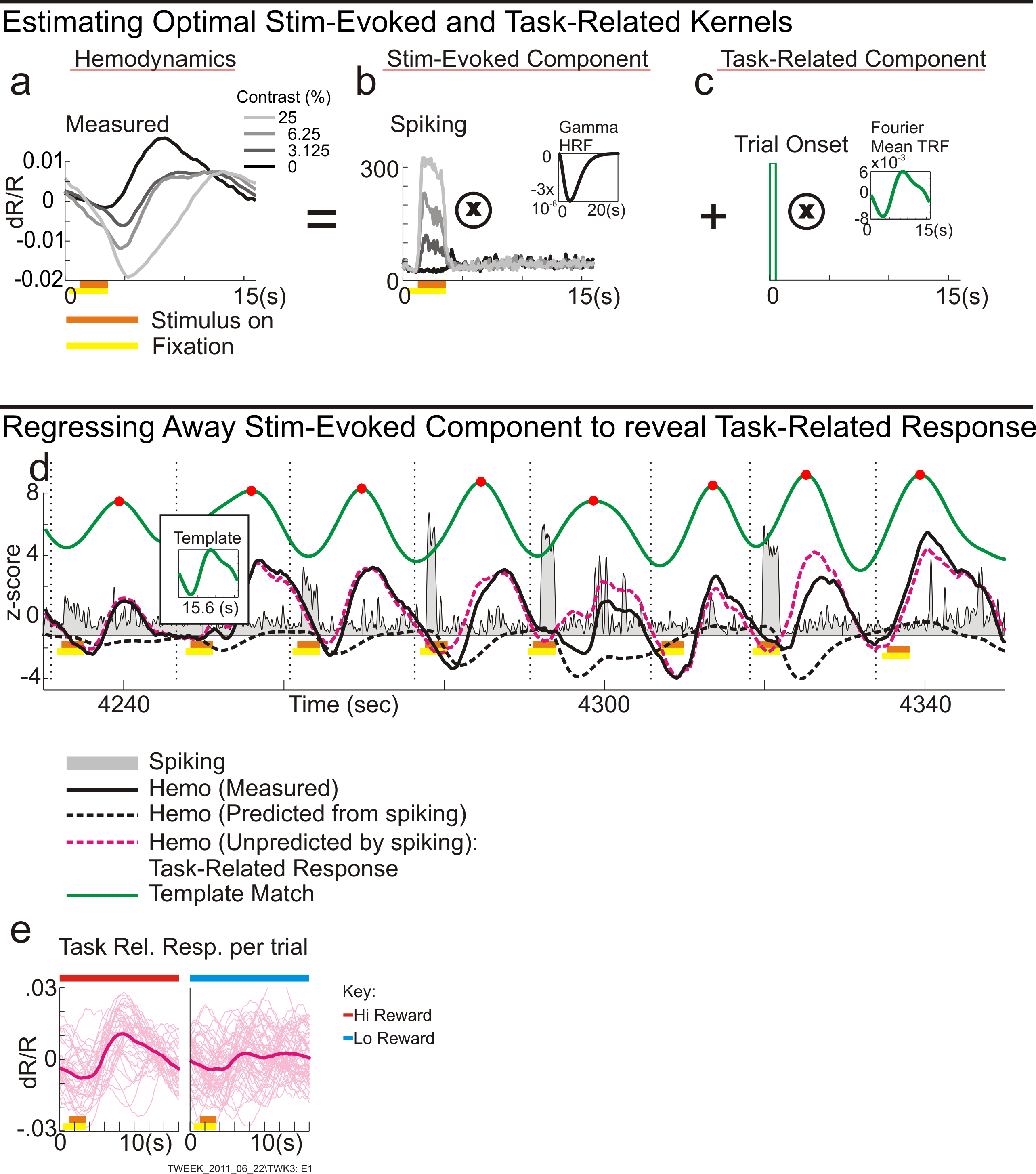

Supplement: S5 Fig — (a-c) Estimating optimal fitted parameters (see Methods, Eqs 4–6). (a) The mean hemodynamic response per stimulus contrast (see key), averaged across trials. The response is modeled as the sum of the stimulus-evoked component (b) and the task-related component (c). The stimulus-evoked component is modeled as the convolution (⊗) of the measured spiking with a gamma-variate HRF kernel (inset). The mean task-related component is modeled as the convolution of delta functions at trial onset with a “Mean TRF” kernel comprising a partial Fourier sum with its fundamental at the trial period (inset). Earlier work showed that the fundamental and the first harmonic terms of the Fourier series are adequate. Insets show the optimal fitted gamma-variate HRF (in b) and optimal mean TRF (in c), respectively. (d) Set of traces illustrating the process of estimating the residual task-related response and then estimating its timing and amplitude per trial by matching to a template (see Methods, Eqs 7 and 8). “Spiking,” “Hemo”: full measured responses, individually z-scored. “Hemo (predicted from spiking)” is the convolution of the spiking response with the optimal fitted HRF (b, inset). Subtracting this from the measured hemodynamic response gives the residual “Hemo (Unpredicted by spiking),” which we defined to be the task-related response. The moving-window dot product of this residual with the template (the optimal fitted mean TRF [c), inset]) gives the “Template Match” (shifted up for visibility). Timing and amplitude of task-related responses, per trial, are defined to be the location and height of each Template Match peak, as for the dark-room task. Showing a section of the full experiment of 483 trials (122 correct). (e) Set of all residual task-related responses, converted back from z-scored values, separated into trials grouped by reward size. The same data are shown in Fig 5A. HRF, hemodynamic response function; TRF, task-related function. (TIF) [file pbio.3000080.s005.tif]

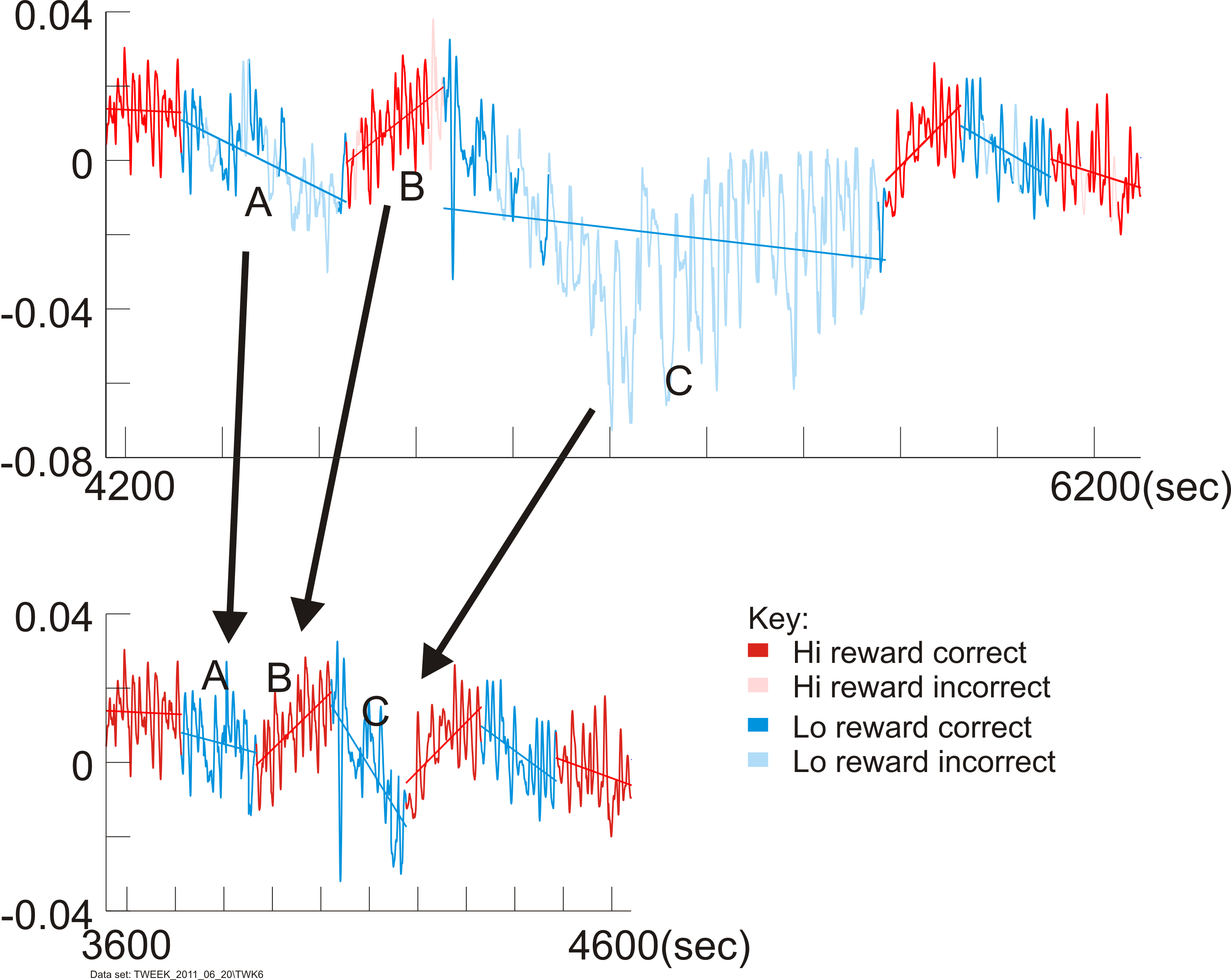

Supplement: S6 Fig — Comparing regression lines through alternating blocks of high and low reward, before (top panel) and after (bottom panel) removing error trials. Color coding for high (red) and low reward (cyan) is the same as in the main text. Error trials are indicated in lighter colors and are grouped with the reward block corresponding to the immediately preceding correct trial. Straight lines show regression fits. Letters (“A,” “B,” “C”) and arrows identify corresponding blocks. Blocks A and B contain individual or short stretches of error trials. C includes a roughly 400-second stretch during which the animal napped. The time axis has the same scale for both top and bottom panels, with time 0 indicating the start of the experiment; the bottom concatenates time points for correct trials. Six consecutive blocks are shown from an experiment comprising 47 blocks (482 correct trials of 684 total). (TIF) [file pbio.3000080.s006.tif]

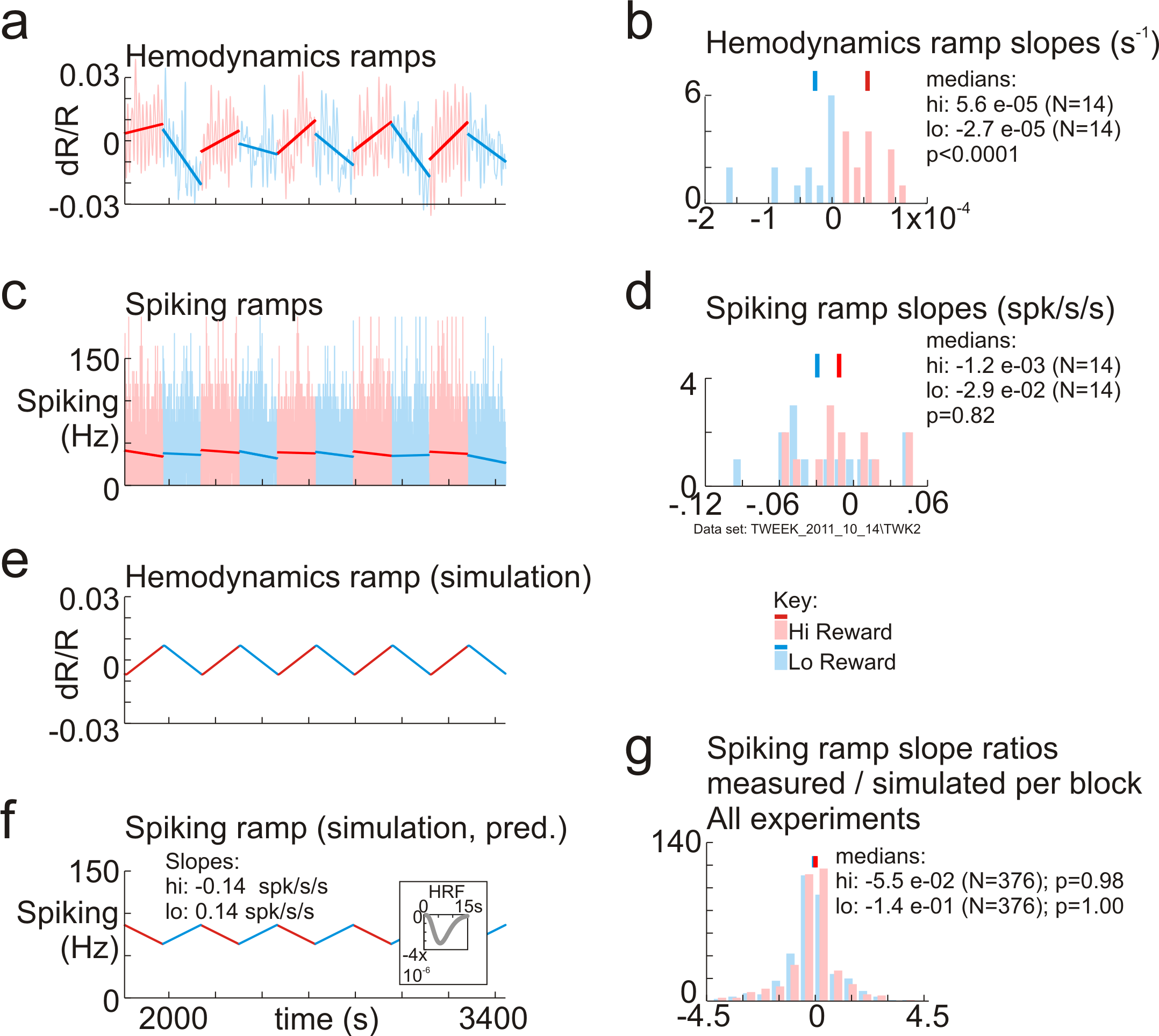

Supplement: S7 Fig — (a, c) Hemodynamics and spiking, respectively, showing correct trials from alternating blocks of high and low reward. Lines show regression fits per block (same data set as Figs 2 and 3). (b, d) Histograms with slopes of regression fits from (a), (c). (e) Simplified simulation of slow mean hemodynamic responses: triangle wave of matching period, with slopes equal to the median (absolute) slopes of the regression lines in (a) (= 4.1 × 10^−5^/second). (f) Simulated spiking response that generates the model hemodynamic response in (e) on convolving with the visually stimulated HRF for this recording site (see “HRF kernels,” S1 Fig; also, Methods). Measured spiking regression slopes (d) are only about 4× weaker than those in the simulation; but they do not alternate in sign with reward size. (g) Distributions of the ratios of measured spiking regression slope per block to the slope of the corresponding simulation, as in (e), (f), across all experiments (N = 752 blocks of 10 trials each, 376 blocks/reward size; from N = 11 experiments with electrode recordings and at least 10 blocks per reward size). p-Values test for the probability of the distributions being centered on zero (bootstrap, 10,000 resamples) (data in S27 Data). HRF, hemodynamic response function. (TIF) [file pbio.3000080.s007.tif]

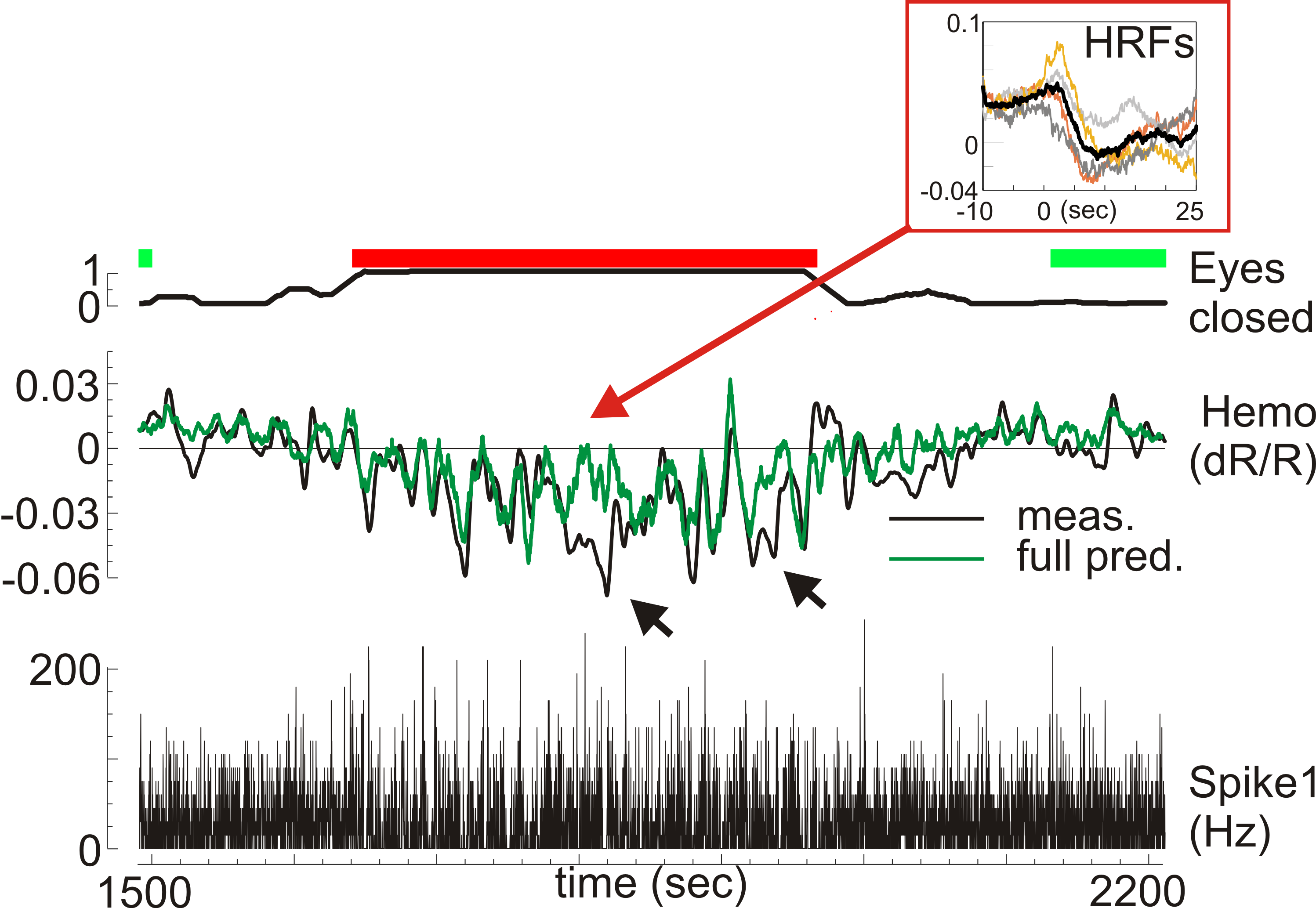

Supplement: S8 Fig — The full prediction here matches the measured response reasonably well except for a few locations with large mismatches (black arrowheads; compare with the same locations in Fig 8A). The overall goodness of fit R2 = 0.76, averaged over this rest epoch, is worse than for the fit with an intercept (R2 = 0.94; see Fig 8A, text). The inset shows HRFs from the deconvolution windows covering this rest epoch, as in Fig 8A; colors identify corresponding HRFs for the two fits. HRF, hemodynamic response function. (TIF) [file pbio.3000080.s008.tif]
